# Psychometric Properties of the Standardised Instruments that are Used to Measure (Pragmatic) Intervention Effects in Autistic Children: A Systematic Review

**DOI:** 10.1177/23969415251341251

**Published:** 2025-05-07

**Authors:** Tatiana Pereira, Ana Cláudia Lopes, Ana Margarida Ramalho, Marisa Lousada

**Affiliations:** 1 RISE-Health, 56062 University of Aveiro, Aveiro, Portugal; 2 Center of Linguistics of the University of Lisbon (CLUL), Faculty of Letters, 37809 University of Lisbon, Lisboa, Portugal; 3 School of Health Sciences of Alcoitão (ESSA), Alcabideche, Lisbon, Portugal; 4 School of Health Sciences (ESSUA), 56062 University of Aveiro, Aveiro, Portugal

**Keywords:** Autism, pragmatic intervention, outcome measures, standardized instruments, psychometric properties

## Abstract

**Background and aims:**

Pragmatic language difficulties can negatively influence the learning, socialization, and mental health of children diagnosed with autism spectrum disorder (ASD). Several studies have sought to determine the effects of interventions, including competencies to help these children use language for social purposes. However, are the instruments used to measure the results of the interventions appropriate and psychometrically adequate? This systematic review aims to analyze the psychometric properties of the standardized instruments that are used to measure the effects of interventions addressing (not exclusively, but also) pragmatic language competencies for autistic children.

**Method:**

Following the Preferred Reporting Items for Systematic Reviews and Meta-Analyses guidelines, systematic literature research was carried out in four electronic indexing databases: CENTRAL, PubMed, Web of Science, and Scopus.

**Results:**

A total of 49 studies from 2005 to 2023 were included and 19 standardized instruments were identified.

**Conclusions:**

After analyzing the instruments psychometric properties, the results indicated that all present some evidence of validity and reliability, but none report responsiveness. Implications: Given the impact that an instrument can have on analyzing the effects of an intervention, this study highlights the importance of considering not only the validity and reliability of an instrument but also responsiveness as a psychometric property, and the need to better describe the rationale for the outcome measures and specify what abilities are being targeted and measured. This will accurately guide future research and improve clinical decision-making around ASD.

## Introduction

Autism spectrum disorder (ASD) is a lifelong neurodevelopmental condition characterized by persistent difficulties in social communication and social interaction and restricted repetitive behaviors, activities, or interests (American Psychiatric Association, 2022).

Over the last 2 decades, the Centers for Disease Control and Prevention have reported a noticeable increase in the number of children aged eight diagnosed with ASD across the United States. According to recent data collected in 2020, it was estimated that one in 36 children were diagnosed with ASD (Maenner et al., 2023) which represents an increase of approximately 20% since 2018, when the incidence was one in 44. Current evidence from epidemiological studies in Europe also supports an increase in ASD prevalence (Bougeard et al., 2021).

The severity of the symptoms varies extensively and leads to a multitude of clinical presentations. Furthermore, autistic children often present comorbid psychiatric conditions, which also increase clinical heterogeneity (Bougeard et al., 2021). Difficulties in using language for social purposes (pragmatics) are a communication core feature of autistic children, although formal language competencies may or may not be impaired (American Psychiatric Association, 2022). Considering this heterogeneity, several interventions are designed holistically and address several competencies (including pragmatic language), which, in turn, is also reflected in the outcome measures used to analyze the effectiveness of those interventions.

According to Prutting and Kirchner (1987), initial definitions of pragmatics involved three aspects that are mastered synchronously: verbal (e.g., speech acts, topic selection, introduction, and maintenance; turn-taking response and initiation), paralinguistic (e.g., prosody, fluency), and nonverbal (e.g., gestures, facial expression, eye gaze). More recently, Parsons et al. (2017) suggested that this definition has been extended to embrace social, emotional, and communicative aspects of language, which reflect an understanding that social and emotional skills are interconnected with pragmatics. This definition of pragmatic language will be used for this review.

Considering the long-term impact that pragmatic language difficulties may have on autistic children, early, effective, and evidence-based interventions are crucial (Cummings, 2017). Several intervention approaches, which vary in terms of their philosophical foundations and application, have been promoted and used to support expressive and receptive language development in autistic children. These include behavioral interventions; developmental approaches; naturalistic developmental behavioral interventions; sensory-based interventions; animal-assisted interventions; technology-based interventions; classroom-based interventions and cognitive-behavioral interventions (Sandbank, Bottema-Beutel, Crowley, Cassidy, Dunham et al., 2020).

Recent research has focused on interventions targeting specific social communication skills in children with ASD and several studies have examined the effectiveness of interventions addressing, for example, joint attention and joint engagement (Kasari et al., 2014; Landa et al., 2011; Roberts et al., 2023).

To facilitate comparison across studies, researchers have explored the use of standardized measures such as the Brief Observation of Social Communication Change (BOSCC) to assess broader social communication skills (Swain et al., 2024). Swain et al. (2024) suggest that the BOSCC could provide a consistent measurement method across various intervention models, effectively assessing the impact on overall social communication skills, but it might not fully capture the effects of brief interventions focusing on proximal outcomes.

Although many interventions were reported in the literature, there was not a systematic review that included most of the evidence produced and that allowed clinicians to make evidence-based decisions. Parsons et al. (2017) published a systematic review with meta-analysis of pragmatic language interventions for autistic children. Following a broad definition of pragmatic language, the authors considered interventions that addressed: preverbal pragmatic language, introduction and responsiveness, nonverbal communication, social-emotional attunement, executive functions, and negotiation. The review included 22 studies and 20 pragmatic language interventions. Some of the included interventions (e.g., guided through music, sports or animals), aimed to study their effectiveness on self-regulation, adaptative and motor behaviors, beyond the socialization and communication domains. As a result, a wide range of assessment instruments were used. Some were standardized, others were not. Parsons et al. (2017) reported that, across the included studies, the measurement of the results often assessed the effects in the context in which the intervention was administered or through a decontextualized assessment instrument, so conclusions were not drawn about the generalization of skills following these interventions. The authors emphasize the importance of having instruments that capture the complex nature of social interactions so that researchers and clinicians can measure changes after intervention, as well as skills maintenance and generalization (Parsons et al., 2017). This highlights the need for researchers to consider including assessment instruments in their investigations that capture behavioral observations of pragmatic language skills in various contexts.

As part of a larger meta-analysis (Project AIM), Sandbank, Bottema-Beutel, Crowley, Cassidy, Feldman, et al. (2020) sought to determine whether existing interventions significantly improve the language skills of young autistic children, considered broadly and more specifically according to a subtype of receptive, expressive, and compositive language outcome, and to evaluate the extent to which summary effects varied by intervention, participant and outcome characteristics. The authors found evidence that intervention could facilitate improvements in language outcomes for young autistic children. Effects were larger for expressive and composite language outcomes, for children with initially higher language abilities, and for interventions implemented by clinicians or by caregivers and clinicians combined. However, the analysis of study quality indicated that study designs need to be improved in future research to draw strong conclusions about the effects of interventions on the language outcomes of young autistic children. The authors also mentioned that the borderline significance of some results tempers study conclusions regarding intervention effectiveness and corresponding moderators (Sandbank, Bottema-Beutel, Crowley, Cassidy, Feldman et al., 2020). Despite their importance, the measurement properties of the outcome measures were not considered, so it is unknown whether the outcome measures’ psychometric adequacy may have contributed to the results achieved.

One difficulty in interpreting research findings is the multitude of measurement instruments used to collect evidence of progress and outcomes. The instruments are of varying relevance and have limited evidence of their measurement properties when used with autistic children. Additionally, when selecting outcome measures for interventions, it is essential to consider the distinction between proximal (immediate, direct changes targeted by the intervention) and distal (broader outcomes like improved quality of life or long-term benefits) effects. The choice of outcome measures should align with these targets, with proximal measures being specific and highly responsive to short-term changes and distal measures capturing broader constructs over extended periods (McConachie et al., 2015).

Measuring change is essential to understanding the effectiveness of interventions (Polit, 2015) and the choice of the outcome measurement instrument is a critical decision to guide research and clinical practice accurately. For this reason, several criteria must be considered when choosing an outcome measurement instrument, including the psychometric properties (Denman et al., 2017), that must be determined and found to be adequate.

According to the consensus-based standards for the selection of health measurement instruments (COSMIN) taxonomy of measurement instruments, three quality domains are distinguished: validity, reliability, and responsiveness (Mokkink et al., 2010).

Domain *validity* includes three measurement properties: content validity, criterion validity, and construct validity. Content validity refers to the degree to which the content of an instrument is an adequate reflection of the construct to be measured and it contains one aspect: face validity (Mokkink et al., 2010). Although it can be assessed quantitatively, through the content validity index (Yusoff, 2019), content validity is usually assessed by carefully checking the measurement method against the conceptual definition of the construct (Price et al., 2017). Criterion validity includes predictive validity (the degree to which the result of a test (or measurement) predicts the future behavior of the individual) and concurrent validity (the degree to which a new method correlates with an existing, valid one). Construct validity is the degree to which the scores of an instrument are consistent with hypothesis based on the assumption that the instrument validly measures the construct to be measured. It contains three aspects: (a) structural validity, which concerns the internal relationships, and (b) hypothesis-testing and (c) cross-cultural validity, which both concern the relationships to scores of other instruments or differences between relevant groups (Mokkink et al., 2010). Construct and criterion validity can be determined by calculating correlations (Polit & Yang, 2016).

The domain *reliability* contains the measurement properties of internal consistency, reliability, and measurement error (Mokkink et al., 2010). Internal consistency is usually reported by Cronbach's α (Andresen, 2000; Salter et al., 2005). Pearson's product-moment correlation coefficient (r), Spearman's rank correlation (r), kappa statistic (k), or intraclass correlation coefficient (ICC) are frequently employed to analyze reliability (Polit & Yang, 2016). Fleiss (1986) proposed a classification for strength reliability based on the ICC as follows: below 0.40 is poor; between 0.40 and 0.75 is fair to good and above 0.75 is considered excellent. Regarding Cronbach's alpha, if it is above 0.90 it is considered excellent; if between 0.80 and 0.90 it is characterized as good; reasonable if between 0.7 and 0.8 and weak if between 0.6 and 0.7 (Tavakol & Dennick, 2011).

The domain *responsiveness* contains only one measurement property, which is also called responsiveness. Several parameters proposed in the literature to assess responsiveness were considered inappropriate by Mokkink et al. (2010) because they failed to align with the conceptual definition of responsiveness or did not provide valid evidence for their assessment. These include effect sizes, standardized response means, Norman's responsiveness coefficient, paired t-test, and Guyatt's responsiveness ratio. On the other hand, correlations between change scores or the area under the receiver operator curve were considered appropriate methods for measuring responsiveness. This measurement property is particularly important when instruments are used to measure outcomes of interventions and should influence decision-making (if an instrument does not show evidence of responsiveness, this should be considered and assumed when choosing an outcome measurement instrument; Polit, 2015).

Of all the linguistic dimensions, pragmatic language is the most highly dynamic and context-dependent, which presents a challenge for the assessment. Given its complex nature, pragmatic language can be particularly difficult to assess through standardized instruments directly applied to the children (Shipley & McAfee, 2021; Tager-Flusberg et al., 2009), although this is the most frequent method used to assess children's language (Binns & Cardy, 2019). Other methods, such as parent/teacher reports and structured/direct observation have been used to assess pragmatic language skills (Norbury, 2014; Pereira, Ramalho, Sá Couto, & Lousada, 2025). However, the bias introduced with the use of parent/teacher-rated measures highlights the need for further development around pragmatic language measurement. Instruments that capture the complex nature of social interactions are needed so that researchers and clinicians can obtain unbiased measurements of the effects of interventions addressing pragmatic language competencies, their generalization, and maintenance over time (Jensen de López et al., 2022; Parsons et al., 2017; Pereira & Lousada, 2023; Pereira, Ramalho, & Lousada, 2025).

For an intervention's results to be reliable and useful for guiding research and clinical practice, the outcome measurement instruments must measure what they are intended to (validity), provide stable results under different conditions (reliability), and be responsive to change over time (responsiveness). If information on psychometric properties is missing or inadequate, concerns may arise about the results’ accuracy and their use in making crucial clinical decisions (Friberg, 2010). A recent systematic review conducted by Pereira and Lousada (2023) analyzed the psychometric properties of the instruments that were used to determine the effects of pragmatic interventions for children with developmental language disorder. The authors reported that across the included studies, all outcome measurement instruments present some evidence of validity and reliability, but none reported responsiveness. Additionally, the instruments reviewed were not used for their original purpose, and some of them were not related to the content of the interventions, which may have contributed to the absence of statistically significant differences in the intervention studies. This highlights the importance of analyzing the effectiveness of interventions carefully and based on several criteria. Pereira and Lousada (2023) concluded that considering the importance of this topic for research and clinical practice, future studies should explore the adequacy of the outcome measures used to analyze the effectiveness of an intervention in other neurodevelopmental conditions, such as ASD.

A comprehensive synthesis was not found examining reliability, validity, and responsiveness within the context of interventions addressing (not exclusively, but also) pragmatic language competencies for autistic children. Focusing on this knowledge gap will guide both clinical practice and research toward more effective and accurate outcome measurement.

Thus, this systematic review aims to analyze the psychometric properties of the standardized instruments that are used to measure the effects of interventions addressing (not exclusively, but also) pragmatic language competencies in autistic children, following the broad definition of pragmatic language described in Parsons et al. (2017) and also used in Pereira and Lousada (2023) systematic review. This research is intended to analyze if the standardized instruments used to measure the effects of interventions—addressing (not exclusively, but also) pragmatic language competencies—for autistic children are appropriate and psychometrically adequate to detect changes over time. Specifically, the review has three research questions: (a) What is the validity of the instruments? (b) What is the reliability of the instruments? (c) How responsive are the instruments in detecting changes over time?

## Method

Prior to the development of this systematic review, searches in PubMed and the International Prospective Register of Systematic Reviews (PROSPERO) were conducted to exclude the existence of protocols or reviews with the same purpose as this one. No similar studies or protocols were found, so a review protocol was written and registered at PROSPERO (Registration No. CRD42022315927).

This systematic review followed the Preferred Reporting Items for Systematic Review and Meta-Analysis (PRISMA) guidelines (Page et al., 2021). A completed PRISMA checklist is provided in Appendix A.

### Information Sources

A systematic literature search was conducted in four electronic indexing databases: CENTRAL, PubMed, Web of Science, and Scopus. The first search was conducted on the 31st of May 2022 and then repeated on the 31st of December 2022. The authors have received weekly automatic updates on potential eligible articles (based on the research previously carried out in databases) up to December 2023. The reference lists of the included studies and previous published systematic reviews of pragmatic language interventions were also searched to identify other potentially eligible studies and ensure literature saturation. Authors were contacted to obtain full texts when needed.

### Search Strategy

The following terms were searched: “autism spectrum disorder” AND “pragmatic language” AND (“standardized instruments” OR assessment OR intervention OR effects OR “outcome measures”). Also, the same search strategy and combination of Boolean operators were used for older terminologies (e.g., “autism”; “Asperger syndrome”; “autistic”; “autistic disorder”; “pervasive developmental disorder not otherwise specified”; “Rett syndrome,” and “child disintegrative disorder”). The database filters, when available, were applied to limit the results to peer-reviewed articles written in English from 2005 to 2023 study design (randomized controlled trial or controlled trial), and participants’ ages (under 18). The decision to use a time window was based on previous results of Parsons et al. (2017). The goal was to include all pragmatic interventions that used standardized instruments as outcome measures, knowing that probably no studies prior to 2005 could be included. Regarding study design, experimental or quasi-experimental designs were only included because the risk of bias (RoB) is more controlled and provides more reliable evidence. Considering participant age, although the diagnosis can be made very early, the age of the diagnosis varies worldwide (Crasto et al., 2024), which is why no minimum age was set. The full search strategy and the filters applied for each database can be consulted in Appendix B. The first and fourth authors independently searched each database and reached an agreement of 100% for search results.

### Eligibility Criteria

Considering the purpose of this systematic review, randomized controlled trials, and nonrandomized controlled trials (quasi-experimental studies) that addressed (not exclusively, but also) pragmatic language skills for children under the age of 18 with an ASD diagnosis, were included. This includes interventions that were not mainly designed as pragmatic interventions but whose aim was to improve some skills related to the use of the language. Studies that included autistic children, but not exclusively, were also included. Nonexperimental studies, case studies, case series, review articles, clinical notes, magazines, news, research protocols, thesis, reports, dissertations, abstracts, communications, posters, letters to the editor, guidelines, statements, position papers, unpublished work, books, and studies published in the form of book chapters were excluded to focus on high-quality, peer-reviewed and methodologically sound studies. Qualitative studies were excluded considering the purpose of this review. Pilot studies and pharmacological treatments were also excluded. Additionally, to be considered eligible for this review, the studies had to include at least one standardized instrument as an outcome measure (with one or more subtests to assess pragmatic language skills or to examine related competencies, such as communication or social skills). Studies without standardized instruments as outcome measures were excluded.

### Study Selection

After grouping the searched reports using EndNote 20 (version 20.5.0.18631), duplicates were automatically removed. Subsequently, the titles, abstracts, and keywords of the remaining reports were screened, and the first and fourth review authors individually applied the eligibility criteria. Then, the full text of the potentially eligible articles was carefully and independently read by the first and fourth authors to ensure the reliability of the eligibility criteria. Any disagreements between the authors were discussed and resolved by consensus with a third researcher (third author). All the records that met the eligibility criteria were included. The standardized instruments used in the included studies and related to pragmatic language skills (even with just some items or subtests) were all considered, regardless of whether psychometric properties data was available or not.

### Data Collection

The first author retrieved data from the included studies (authors and year, study design, aims/research question, participants’ characteristics, intervention, and outcome measures) and it was independently analyzed by the fourth author. Any disagreements were resolved through discussion.

### RoB Assessment

The RoB in each study was assessed independently by the first and second authors. Considering that both randomized and nonrandomized controlled trials were included, the RoB 2 was used to assess the RoB in randomized controlled trials, and the RoB In Nonrandomized Studies of Interventions (RoBINS-I) was used to determine the RoB in nonrandomized controlled trials. These are two of the most recommended scales for evaluating the RoB in intervention studies.

Five domains were assessed through RoB-2: (a) randomization process, (b) deviations from the intended intervention, (c) missing outcome data, (d) measurement of the outcome, and (e) selection of the reported result (*Cochrane Handbook for Systematic Reviews of Interventions*, 2023). The Excel macro form for RoB-2 was used to input the answers that authors gave to signaling questions. An algorithm estimated the overall risk of the bias according to the results for each domain as low-risk, some concerns, or high-risk (note that it only takes one domain to present a high RoB for the overall to be the same). First, each of the authors entered their answers into different Excel files, and then they were combined to check discrepancies and reach consensus.

The domains assessed through ROBINS-I were (a) bias due to confounding, (b) bias in the selection of participants into the study, (c) bias in the classification of interventions, (d) bias due to deviations from intended interventions, (e) bias due to missing data, (f) bias in the measurement of the outcome and (g) bias in the selection of the reported result (*Cochrane Handbook for Systematic Reviews of Interventions*, 2023). The authors answered signaling questions and then estimated the overall risk of the bias according to the results for each domain as low, moderate, serious, or critical. The RoB plots, for both scales, were drawn using the *robvis* web app (McGuinness & Higgins, 2021).

Additionally, the interrater agreement of the quality assessment performed by two of the authors was evaluated using Cohen's kappa before the discrepancies were checked and a total consensus was reached. The value of Cohen's kappa ranges from zero to one, illustrating a slight (≤0.2), fair (0.21–0.4), moderate (0.41–0.6), substantial (0.61–0.8), or almost perfect (≥0.81) agreement.

### Psychometric Properties

For this review, the following psychometric property domains (previously addressed in the introduction section of this article) were considered: validity, reliability, and responsiveness. The authors sought validation information provided by the outcome measure developers, publishers or other research teams, and therefore the measurement properties were retrieved from the assessment manuals or published papers.

## Results

### Study Selection

**Figure 1.**
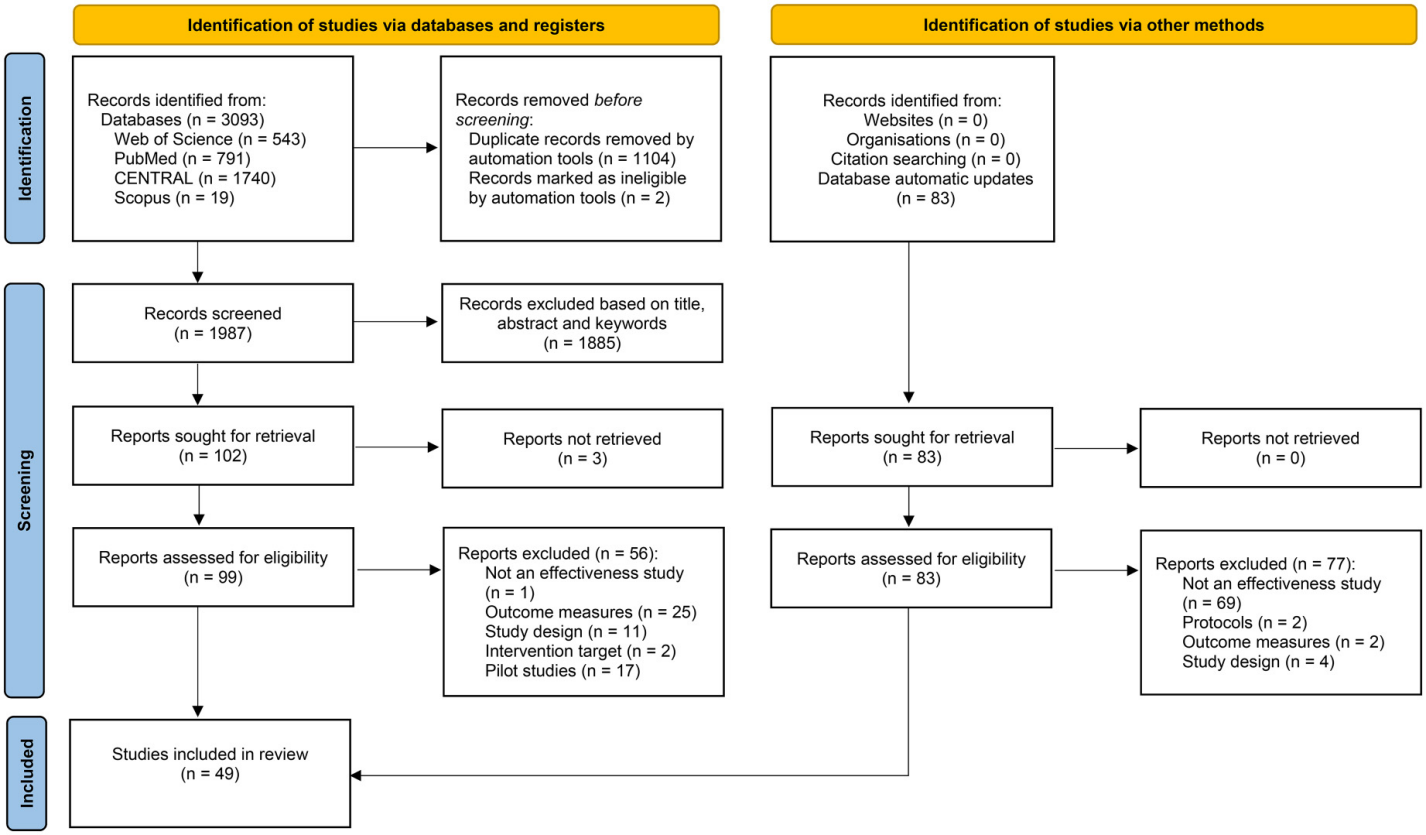
PRISMA Flow Diagram.

The systematic database search identified 3,093 publications. Two papers were marked as ineligible by automation tools (retracted) and 1,104 were duplicates. After removing duplicates, the title, abstract, and keywords were screened for 1987 papers. From these, 102 papers were full-text screened to accurately assess eligibility according to the established criteria. Three papers were not retrieved. The weekly automatic updates from December 2022 until December 2023 suggested an additional 83 papers. From the total 182 papers assessed for eligibility, 128 were excluded. A total of 49 papers were included. Figure 1 presents the PRISMA flow diagram.

PRISMA = Preferred Reporting Items for Systematic Review and Meta-Analysis.

### Study Characteristics

From the 49 included papers, 43 were randomized controlled trials and six were nonrandomized controlled trials. The participants’ age ranged from 18 months to 18 years. The interventions were delivered by parents or professionals in schools, clinics or at home.

The characteristics of the 49 included papers can be consulted in Tables S1 and S2 in the Supplemental Material.

### RoB Across Studies

The first and second authors performed the RoB assessment, and the Cochrane RoB tools for randomized (RoB-2) and nonrandomized (ROBINS-I) studies were used. The agreement between the two authors (before the discrepancies were checked) was substantial (k = 0.69, *p* < .001). A final and total consensus was reached.

**Figure 2.**
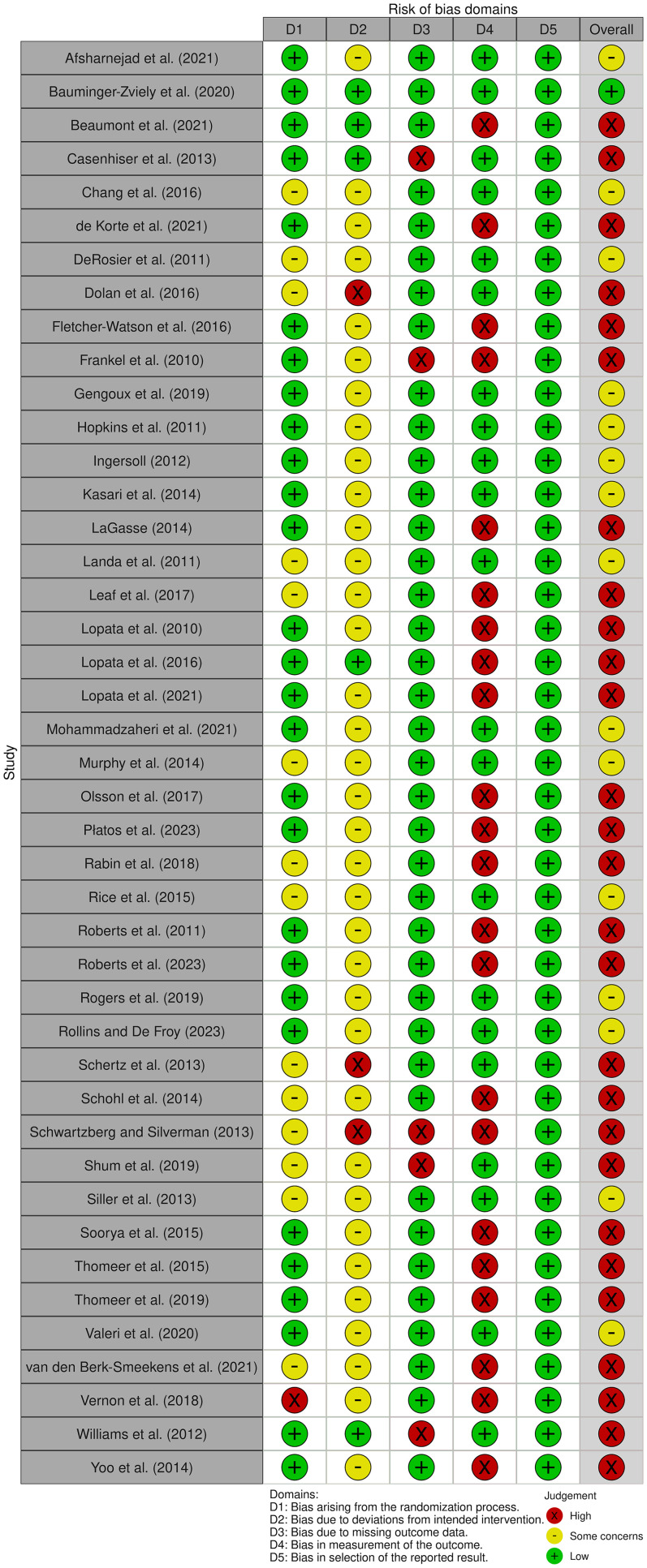
Risk of Bias Assessment of the Included Randomized Controlled Trials Using RoB-2.

**Figure 3.**
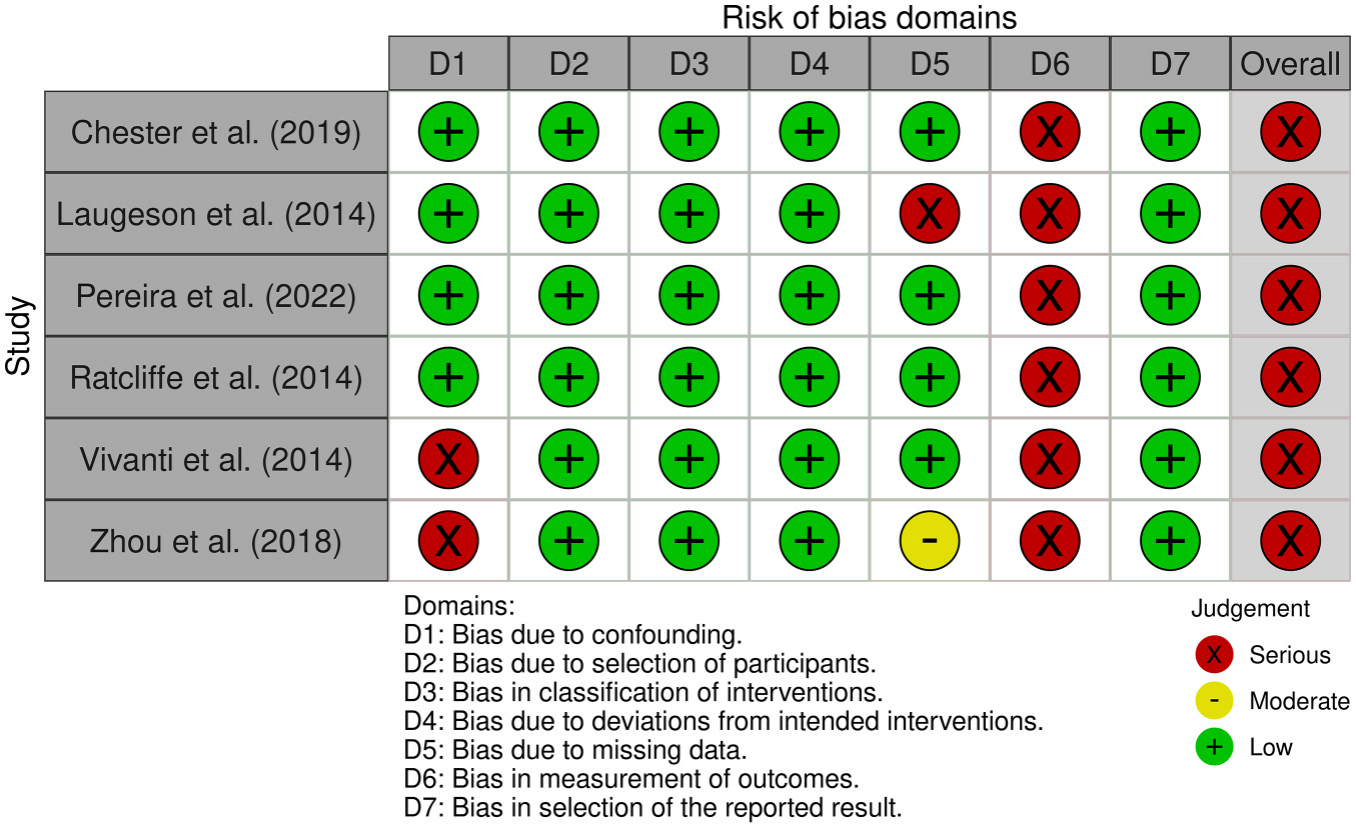
Risk of Bias Assessment of the Included Nonrandomized Controlled Trials Using RoBINS-I.

More than 50% of the randomized controlled trials studies assessed with RoB-2 present an overall high RoB. Specifically, 27 were judged at high RoB; 15 studies were judged at moderate RoB, and just one study was judged at low RoB. All the nonrandomized controlled trials assessed with ROBINS-I present an overall RoB judged as serious. Figures 2 and 3 illustrate the plots of the quality assessment results for randomized and nonrandomized controlled trials, respectively.

RoB-2 = risk of bias-2.

RoBINS-I: risk of bias in nonrandomized studies of interventions.

### Included Studies and Outcome Measures

Among the 49 papers included, 19 standardized outcome measurement instruments (the following versions of the instruments’ original forms were not considered for the counting) were identified: Social Responsiveness Scale (SRS) and SRS-Second Edition (SRS-2; Constantino, 2012; Constantino & Gruber, 2005); Vineland Adaptative Behavior Scales (Vineland) and Vineland Adaptative Behavior Scales-Second Edition (Vineland-II; Sparrow et al., 1984, 2005); Social Skills Questionnaire (Spence, 1995); Preschool Language Scale-Fourth Edition (PLS-4) and PLS-Fifth Edition (PLS-5; Zimmerman et al., 2011, 2006); Mullen Scales of Early Learning (MSEL; Mullen, 1995); Social Skills Rating System and Social Skills Improvement System (Gresham & Elliott, 1990, 2008); Test of Adolescent Social Skills Knowledge (TASSK) and TASSK-Revised (TASSK-R; Laugeson & Frankel, 2006, 2010; Laugeson et al., 2012); Communication and Symbolic Behavior Scales Developmental Profile (CSBS-DP; Wetherby & Prizant, 2002); Autism Diagnostic Observation Schedule (ADOS), ADOS-Generic (ADOS-G), ADOS-Second Edition (ADOS-2) and ADOS Toddler Module (Gotham et al., 2009; Lord et al., 2012a, 2012b, 2000, 1999); Comprehensive Assessment of Spoken Language (CASL; Carrow-Woolfolk, 1999); Children's Communication Checklist (CCC) and CCC-Second Edition (CCC-2; Bishop, 2003, 2006); Test of Pragmatic Skills (Shulman, 1986); (New) Reynell Developmental Language Child (Edwards et al., 1997, 2011); Teste de Linguagem–Avaliação da Linguagem Pré-Escolar (TL-ALPE; Mendes et al., 2014); Escala de Avaliação de Competências Comunicativas (EAC; Seabra et al., 2021); Bayley Scales of Infant and Toddler Development–Third Edition (Bayley-III; Bayley, 2005); Brief Observation of Social Communication Change (BOSCC; Grzadzinski et al., 2016) and Autism Social Skills Profile (ASSP; Bellini & Hopf, 2007).

A list of the instruments and the corresponding papers that used them can be seen in Table 1.

**Table 1.** A List of the Instruments and the Corresponding Papers that Used Them.

Standardized instruments	Studies
SRS and SRS-2 (Constantino, 2012; Constantino & Gruber, 2005)	Afsharnejad et al. (2021), de Korte et al. (2021), DeRosier et al. (2011), Gengoux et al. (2019), LaGasse (2014), Laugeson et al. (2014)*, Leaf et al. (2017), Lopata et al. (2010, 2016, 2021), Olsson et al. (2017), Płatos et al. (2023), Rabin et al. (2018), Rice et al. (2015), Schohl et al. (2014), Shum et al. (2019), Soorya et al. (2015), Thomeer et al. (2015, 2019), van den Berk-Smeekens et al. (2021), and Vernon et al. (2018)
Vineland and Vineland-II (Sparrow et al., 1984, 2005)	Bauminger-Zviely et al. (2020), de Korte et al. (2021), Gengoux et al. (2019), Roberts et al. (2011), Rogers et al. (2019), Schertz et al. (2013), Schohl et al. (2014), Vivanti et al. (2014)*, Williams et al. (2012), and Yoo et al. (2014)
SSQ (Spence, 1995)	Beaumont et al. (2021)
PLS-4 and PLS-5 (Zimmerman et al., 2011, 2006)	Casenhiser et al. (2013) and Roberts et al. (2023)
MSEL (Mullen, 1995)	Chang et al. (2016), Gengoux et al. (2019), Kasari et al. (2014), Landa et al. (2011), Rogers et al. (2019), Schertz et al. (2013), Siller et al. (2013), and Vivanti et al. (2014)*
SSIS (Gresham & Elliott, 2008)	Chester et al. (2019)*, Leaf et al. (2017), Rabin et al. (2018), Ratcliffe et al. (2014)*, Vernon et al. (2018), and Yoo et al. (2014)
TASSK and TASSK-Revised (Laugeson & Frankel, 2006, 2010; Laugeson et al., 2012)	Dolan et al. (2016), Laugeson et al. (2014)*, Płatos et al. (2023), Rabin et al. (2018), Schohl et al. (2014), Shum et al. (2019), and Yoo et al. (2014)
CSBS (Wetherby & Prizant, 2002)	Fletcher-Watson et al. (2016), Landa et al. (2011), Roberts et al. (2023), Rollins and De Froy (2023), and Zhou et al. (2018)*
ADOS, ADOS-Generic, ADOS-Calibrated Severity Score, ADOS-2, and ADOS Toddler Module (Gotham et al., 2009; Lord et al., 2012a, 2012b, 2000, 1999)	de Korte et al. (2021), Fletcher-Watson et al. (2016), Kasari et al. (2014), Rogers et al. (2019), Schohl et al. (2014), Valeri et al. (2020), van den Berk-Smeekens et al. (2021), Vivanti et al. (2014)*, Williams et al. (2012), Yoo et al. (2014), and Zhou et al. (2018)*
SSRS (Gresham & Elliott, 1990)	Frankel et al. (2010), Laugeson et al. (2014)*, Hopkins et al. (2011), Schohl et al. (2014) and Yoo et al. (2014)
CASL (Carrow-Woolfolk, 1999)	Lopata et al. (2010, 2021), Thomeer et al. (2019), and Casenhiser et al. (2013)
CCC and CCC-2 (Bishop, 2003, 2006)	Mohammadzaheri et al. (2014, 2021) and Soorya et al. (2015)
TPS (Shulman, 1986)	Murphy et al. (2014)
(New) RDLS (Edwards et al., 1997, 2011)	Roberts et al. (2011)
TL-ALPE (Mendes et al., 2014)	Pereira et al. (2022) *
Bayley-III (Bayley, 2005)	Ingersoll (2012)
BOSCC (Grzadzinski et al., 2016)	Roberts et al. (2023)
ASSP (Bellini & Hopf, 2007)	Schwartzberg and Silverman (2013)
ABAS (Harrison & Oakland, 2003, 2015)	Shum et al. (2019)
EAC (Seabra et al., 2021)	Pereira et al. (2022) *

*Note.* SRS = Social Responsiveness Scale; Vineland = Vineland Adaptative Behaviour Scale; SSQ = Social Skills Questionnaire; PLS = Preschool Language Scale; MSEL = Mullen Scales of Early Learning; SSIS = Social Skills Improvement System; TASSK = Test of Adolescent Social Skills Knowledge; CSBS = Communication and Symbolic Behaviour Scale; ADOS = Autism Diagnostic Observation Scale; SSRS = Social Skills Rating System; CASL = Comprehensive Assessment of Spoken Language; CCC = Children's Communication Checklist; TPS = Test of Pragmatic Language; RDLC = Reynell Developmental Language Child; STAT = Screening Tool for Autism in Toddlers and Young Children; TL-ALPE = Teste de Linguagem-Avaliação da Linguagem Pré-Escolar; Bayley = Bayley Scales of Infant Development; BOSCC = Brief Observation of Social Communication Change; ASSP = Autism Social Skills Profile; ABAS = Adaptive Behavior Assessment System; EAC = Escala de Avaliação de Competências Comunicativas.

* nonrandomized controlled trials.

### Psychometric Properties of the Outcome Measures

The psychometric properties of the identified standardized instruments are presented in Table 2. It is important to mention that only the psychometric properties of the most recent versions of the instruments identified will be presented and that some of the instruments used were designed to assess other skills and not exclusively social (pragmatic) language. Additionally, since some studies have used Chinese, Dutch, Korean, and Polish versions of instruments (e.g., Płatos et al., 2023; van den Berk-Smeekens et al. (2021); Yoo et al. (2014); Zhou et al. (2018), respectively), it was decided to include the psychometric properties of the English versions in the table, referring whenever possible to studies were the psychometric properties of the instruments in another language can be found.

**Table 2.** Psychometric Properties of the Standardized Instruments Used as Outcome Measures.

	Validity			Reliability			
Instruments and Ages	Content Validity	Construct Aalidity Convergent/Discriminant	Criterion Validity Predictive/Concurrent	Internal Consistency	Test–Retest Reliability	Inter- and Intrarater Reliability	Responsiveness
SRS-2 2;6–adulthood	The content of the SRS-2 is the same as the prior version (SRS), now called the School-Age Form. The additional Preschool and Adult form items were slightly modified to reflect the extended age ranges and informant characteristics (e.g., Self-Report Form). The content areas covered reflect the characteristics of ASD outlined in the *DSM-IV-TR*, including social communication, restrictive interests and repetitive behaviors, and reciprocal social interaction, with a focus on deficits in social reciprocity. Items on the original SRS were reviewed by experts representing various fields including special education, psychology, pediatrics, child neurology and psychiatry, and parents of autistic children.	Confirmatory factor analysis was conducted to examine the model fit of the two-factor structure with the two-symptom clusters measured by the SRS-2. The analysis demonstrated good fit for the two-factor model, including the social communication and interaction domain and the restricted interests and repetitive behavior domain.	Predictive validity *School-age form* Estimated using a Receiver Operating Characteristic (ROC) analysis. The analysis resulted in a sensitivity value of 0.92, and specificity value of 0.92. Concurrent validity *School-age form* Moderate to high correlations were found between other rating scales of social behavior and communication (e.g., CCC). Low to moderate correlations were found with other diagnostic instruments including the ADI-R and the ADOS. *Preschool form* Limited research but one independent study found moderate correlations with the Childhood Autism Rating Scale. *Adult form* No concurrent validity data were reported for the Adult form.	Strong internal consistency was found across gender and age and across clinical subgroups within the clinical sample. The clinical sample involved the School-Age form only and yielded a total reliability coefficient of 0.95.	No test–retest data were collected for the SRS-2.	Interrater reliability For the *School-age* and *Preschool* forms, comparisons were across parent and teacher ratings. Correlations were 0.77 and 0.61, respectively. The *Adult Self-Report* form was compared across various raters including mothers, fathers, spouses, and relatives. Reliability coefficients ranged between 0.61 and 0.92.	NR
Vineland-II 0–90 (interview) 3–21 (teacher form)	Vineland-II was designed to measure four major aspects of adaptative functioning: communication, daily living skills, socialization, and motor skills. Each domain has subdomains each with target behaviors which are deemed important to adaptative functioning.	Discriminant The relationship between Vineland-II and the Wechsler Intelligence Scales for Children-Third Edition (WISC-III) and Wechsler Adult Intelligence Scale-Third Edition (WAIS-III) indicate a near-zero correlation.	The Vineland-II was compared to previous version (Vineland) and correlations ranged between 0.69 and 0.96 across ages and domains/subdomains.	Ranged from 0.89 to 0.92 (ages 10–12).	The test–retest reliability ranged between 0.76 and 0.92 (except for the maladaptative behavior susbcales and index, which have test–retest correlations ranging from 0.74 to 0.98).	Interrater reliability Range between 0.71 to 0.81 across domains, subdomains (except for the maladaptative behavior subscales and index) and ages.	NR
SSQ	Content validity was rated as good. To produce items, the authors reviewed previous research and interviewed parents and teachers. These groups rated the importance of the items; however, no quantitative information is reported.	Construct validity of the parent and self-report forms was rated as good and adequate, respectively.	NR	Internal consistency was excellent for the parent form, with most published alphas > .90, and good for the self-report form (alphas > .080).	NR	NR	NR
PLS-5 From birth to 7 years 11 months	Content was clear, applicable, and pertinent for evaluating the proposed skills.	NR	Concurrent validity Correlation coefficients for the PLS-5 and the CELF Preschoolers-2 scores ranged from medium (0.70) to high (0.82).	The split-half reliability was reported based on the Spearman–Brown formula to estimate internal consistency: 0.91 for the auditory comprehension scale; 0.93 for the expressive communication scale; and 0.95 for the overall score.	The test–retest reliability ranged from 0.86 to 0.95.	NR	NR
MSEL From birth to 68 months	Provides a comprehensive assessment of early learning and cognitive development in young children.	Most scores from measures of language (e.g., PPVT) and adaptive behavior (e.g., Vineland-II) loaded onto the same factor as the MSEL scores, supporting convergent validity by suggesting that these tests all index the same underlying construct.	NR	Total (α = 0.91) Individual scales (ranging from 0.75 to 0.83).	Test–retest reliability for the Mullen is adequate (ranging between 0.71–0.96).	Interrater reliability is strong (ranging from 0.91 to 0.99).	NR
SSRS and SSIS	Content validity was rated as excellent for all three forms. The SSIS is a revision of the Social Skills Rating System (SSRS). All aspects of the SSRS were reviewed by focus groups and suggestions for additional content were made. New items were developed based on a review of the literature, following content guidelines and key terms developed by the authors. Additionally, teachers, parents, and youth rated the importance of each item.	NR	NR	The internal consistency of all three forms was excellent, with alphas typically exceeding .90.	Test–retest reliability was good for each form, with correlations > .80 over intervals exceeding one month in community samples.	NR	NR
TASSK-R	Items are derived from key elements of each of the PEERS^®^ didactic lessons.	NR	NR	Coefficient alpha of .56.	NR	NR	NR
CSBS (6–24 months)	Comprehensively captures various dimensions related to communication and symbolic behavior.	NR	Predictive validity Good predictive validity (McCathren et al., 2000). Concurrent validity The concurrent validity of the CSBS is supported by the moderate to strong correlations between the CSBS developmental profile Infant-Toddler Checklist and behavior sample (*r* = .59 to .67 for the Total) and between the caregiver questionnaire and behavior sample (*r* = .65 to .71 for the Total).	Cronbach's alphas for the composites and total raw scores ranged between .87 and .93.	Ranging from 0.79 to 0.88.	Interrater reliability Ranging from 0.92 to 0.97 for the composites and total score.	NR
ADOS-2 12 months to adulthood	The items allow a comprehensive assessment of the characteristics of an ASD.	NR	Solid predictive validity among ADOS-2 algorithms.	Ranging from α = .87 to α = .92 in the social affect domain and from α = .51 to α = .66 in the restricted and repetitive behaviors domain.	Ranging from 0.83 to 0.87 between modules 1 and 3.	Interrater reliability Ranging from 0.94 to 0.97 between modules 1 and 3.	NR
CASL (3–21)	The tests measure the most representative aspects of each language category for each of six age bands.	Developmental progression of scores, intercorrelations of tests, and factor structures of the indexes show construct validity.	CASL is correlated with other measures of language (TACL-R; OWLS; PPVT; EVT) and cognitive ability (K-BIT).	Above 0.92 for all indexes except receptive index (range from 0.85 to 0.90).	Ranges from 0.65 to 0.95 for the individual tests; from 0.92 to 0.93 for the total composite score and from 0.88 to 0.96 for the indexes form.	NR	NR
CCC-2 (4–16)	The content of the checklist was clear, applicable, and pertinent.	NR	NR	For the 10 subscales, range from 0.66 to 0.80 (Bishop, 2003).	Coefficient reliability range from 0.86 to 0.96 (Bishop, 2006).	Interrater reliability Range from 0.157 to 0.559 for subscales and from 0.360 (GCC) to 0.790 (SIDC) for composite scores (Bishop, 2003).	NR
TPS (3–8)	It captures essential aspects of pragmatic language skills.	NR	NR	α = .79.	Test–retest reliability over 3 weeks: 0.96.	Interrater reliability 0.92.	NR
New RDLS (3;00–7;5)	Updated version of RDLS-III ensures that aligns in terms of both good practice and the latest research.	NR	Concurrent validity Correlation between: - New RDLS comprehension and BPVS III was 0.75. - New RDLS comprehension and TROG II was 0.69. - New RDLS production and BPVS III was 0.63. - New RDLS production and TROG II was 0.63. - New RDLS comprehension and New RDLS production 0.8.	Internal reliability coefficients using the Kuder–Richardson Formula 20 were calculated based on the data collected from all the children. These were 0.95 for comprehension and 0.96 for production.	The test–retest reliability (40 children) coefficients were 0.57 and 0.64 for comprehension and production, respectively.	NR	NR
TL-ALPE (3;6–5;12)	The language skills included are well documented in the literature considering language development and therefore ensured content validity.	Positive and significative correlation between the increase of total scores/subtests and ages.	Correlations with TALC indicate a strong correlation with verbal expression subtest (*r* = .85, *p* < .01) and total score (*r* = .92, *p* < .01). The correlation between TALC and auditive comprehension subtest was 0.63, *p* < .01.	Cronbach's alphas ranging between .82 and .95.	NR	Interrater reliability: 0.95. Intrarater reliability: 0.96.	NR
Bayley-III 1–42 months	Items ensure a comprehensive assessment.			Alpha coefficients greater than or equal to .90.			NR
BOSCC	The BOSCC measures broad social communicative behaviors and others associated with ASD.	Convergent validity Correlations with the MSEL receptive language were significant (*r* = −.35, *p* = .05). Discriminant validity No associations of maternal education or family income with the BOSCC social communication domain (χ2(2) = 1.94, *p* = .38), RRB (χ2(2) = 1.75, *p* = .42) domain or the BOSCC core total (χ2(2) = 1.53, *p* = .47). There was also no association of maternal education and family income with the ADOS-2 CSS (χ2(2) = 3.40, *p* = .18).	NR	Social communication subscale α = .83 Repetitive behavior subscale α = .41.	Test–retest reliability (ICCs) was high: 0.89, 95% CI [0.77, 0.98], for the social-communication domain, 0.79, 95% CI [0.62, 0.96], for the RRB domain, and 0.90, 95% CI [0.81, 0.98], for the core total.	Interrater reliability ICCs ranging from 0.97, 95% CI [0.94, 0.99], to 0.98, 95% CI [0.96, 0.99].	NR
ASSP (6–17)	The ASSP content was inspected by ten experts and appropriate modifications were made based on their suggestions.	NR	NR	α = 0.92	0.904 for the total sample.	NR	NR
ABAS-II (0–89)	ABAS-II is based on a theoretical foundation derived from the American Association of Intellectual and Developmental Disabilities.	NR	The correlation between the General Adaptive Composite (GAC) and: - the Vineland Adaptive Behavior Composite was 0.75 for the teacher/daycare provider form, and 0.84 for the teacher form. - Vineland Adaptive Behavior composite was 0.70.	Reliability scores ranged from 0.97 to 0.99 for the GAC scores, 0.91 to 0.98 for the adaptive domains, and 0.80 to 0.97 for the individual skill areas.	The reliability coefficients were 0.90 for GAC scores, between 0.80 and 0.90 for adaptive scores, and between 0.70 to 0.90 for the individual skill areas.	Interrater reliability Ranged between 0.82 and 0.91 for the GAC, 0.78 and 0.84 for adaptive domains, and 0.70 to 0.82 for the individual skill areas.	NR
EAC (4–8)	The EAC content was inspected by five experts and a content validation index of 1 was reached.	Exploratory factor analysis was conducted through varimax method and showed good construct validity: Kaiser Meyer Olkin = 0.8; Bartlett Test of Sphericity *p* < .001.	NR	Strong internal consistency was found in typical development sample (*n* = 143) and across clinical subgroups (ASD, *n* = 97; DLD, *n* = 83).	Test–retest reliability (ICCs) ranging from 0.94 to 1.00 in typical development sample; from 0.64 to 0.84 in ASD sample and from 0.64 to 1.00 in DLD sample.	Interrater reliability ICCs ranged from 0.40 to 0.86 in DLD sample.	NR

*Note.* NR = not reported; SRS = Social Responsiveness Scale; SRS-2 = Social Responsiveness Scale Second Edition; Vineland = Vineland Adaptative Behaviour Scale; Vineland-II = Vineland Adaptative Behaviour Scale-Second Edition; *DSM-IV-TR* = *Diagnostic and Statistical Manual-Fourth Edition-Text Revision*; SSQ = Social Skills Questionnaire; PLS-5 = Preschool Language Scale-Fifth Edition; CELF = Clinical Evaluation of Spoken Language; CASL = Comprehensive Assessment of Spoken Language; MSEL = Mullen Scales of Early Learning; ADOS = Autism Diagnostic Observation Scale; ADOS-2 = Autism Diagnostic Observation Scale Second Edition; TASSK-R = Test of Adolescent Social Skills Knowledge-Revised; CSBS = Communication and Symbolic Behaviour Scale; SSRS = Social Skills Rating System; Bayley-III = Bayley Scales of Infant and Toddler Development-Third Edition; CCC = Children's Communication Checklist; TPS-Test of Pragmatic Skills; ASSP = Autism Social Skills Profile; SSIS = Social Skills Improvement System; ABAS-II = Adaptive Behavior Assessment System-Second Edition; BOSCC = Brief Observation of Social Communication Change; ICC = Intraclass Correlation Coefficient; GAC = General Adaptive Composite; GCC = General Communication Composite; SIDC = social interaction deviance composite; PEERS = Program for the Education and Enrichment of Relational Skills; PPVT = Peabody Picture Vocabulary Test; BPVS-II British Picture Vocabulary Scale-Second Edition; TROG-II = Test for Reception of Grammar-Version 2; TALC = Teste de Avaliação da Linguagem na Criança; TL-ALPE = Teste de Linguagem-Avaliação da Linguagem Pré-Escolar; RDLS = Reynell Developmental Language Scales; EAC = Escala de Avaliação de Competências Comunicativas; DLD = developmental language disorder.

Concerning validity, concurrent validity (under criterion validity) was analyzed in seven instruments (SRS-2; Vineland-II; PLS-5; CSBS; CASL, New RDLS, TL-ALPE and ABAS-II). These instruments globally reported good concurrent validity when compared to other instruments, which means that they correlate with an existing and valid measure. Predictive validity was analyzed in SRS-2, CSBS, and ADOS-2. Construct validity was addressed for SRS-2, Vineland-II, SSQ, MSEL, CASL, BOSCC, and EAC (see Table 2). Regarding content validity, clear content, applicability, and relevance for assessing the proposed skills were verified in almost all instruments.

Measurement properties of reliability were reported across the identified instruments. Specifically, internal consistency was addressed through all. Cronbach's alpha values suggest that internal consistency ranges from excellent to reasonable for most instruments—this means that the items of each of these instruments present homogeneity and measure the same construct.

None of the standardized instruments reported responsiveness, and therefore nothing can be concluded about the ability to detect changes over time. This aligns with the information provided by Denman et al. (2017) and Pereira and Lousada (2023) who noticed, in their systematic reviews, that no assessment manuals reported studies on responsiveness.

## Discussion

This systematic review aimed to analyze the psychometric properties of the standardized instruments that were used to measure the effects of interventions addressing (not exclusively, but also) pragmatic language competencies for autistic children in order to determine whether they are appropriate and psychometrically adequate. It was not the aim of this review to say whether it is the best practice to use standardized instruments to assess pragmatic competencies, but since they are widely used in the literature and clinical practice, this review was intended to help ensure that evidence-based decisions are made and that the results of an intervention study can be well interpreted, considering the instruments used for that purpose.

Considering the difficulties surrounding the definition of pragmatic language and the fact that in addition to social (pragmatic) difficulties, autistic children also have other characteristics inherent to the diagnosis and frequent comorbidities, which lead to the need for comprehensive and holistic intervention approaches, it was decided to include intervention studies that aimed to address pragmatic language skills, but not necessarily exclusively, in order to capture all the relevant literature. This was also considered in previous systematic reviews (Parsons et al., 2017). The COSMIN taxonomy of measurement instruments was followed, and the validity, reliability, and responsiveness domains of each standardized outcome measurement instrument were investigated.

The methodological quality assessment of the included studies revealed that the RoB was present in all study designs. Considering that the studies included in this review are intervention studies with autistic children under 18 years old, it is to be expected that parents or caregivers are aware of the type of intervention being administered to their children. In addition, several outcome measures aimed to explore whether the intervention results have been generalized to other contexts and related to parents’ or teachers’ reports. The data collected through these types of instruments, although very important, can introduce an RoB in the studies since the evaluators are not usually blind to the intervention given or to the group allocation. As most of the studies in this review included these outcomes and the evaluators were not blind to intervention or group allocation, the RoB was high in the measurement of the outcome domain and, consequently, overall. Previous studies have reported that intervention studies in autistic children often struggle to have adequate blinding to overcome measurement bias. Consequently, bias arising from the outcome measurement significantly affects the quality rating of both randomized and nonrandomized controlled trials (Balian et al., 2021), as was found in this review. This can be mitigated with active treatment control designs or when parents or teachers are assessors but are not aware of the intervention or the child's group assignment.

Nineteen standardized instruments with one or more subtests related to language, communication, or social skills that were used to measure pragmatic intervention effects were analyzed.

Further studies regarding responsiveness are needed but some instruments were identified as currently having better evidence of validity and reliability. Considering the data collected, SRS-2 and Vineland-II present the soundest psychometric evidence with only responsiveness data still to be considered. However, considering Vineland-II, it was not expected to be used to detect changes over time after an intervention since it is a diagnostic assessment tool. On the other hand, it is important to bear in mind that SRS-2 is a parent/teacher report, so it can produce biased results if the intervention or group assignment is not blind to parents and teachers. Therefore, this does not mean that the instruments with the best evidence of validity and reliability are the best instruments for assessing the effects of pragmatic language interventions, as several criteria must be considered when choosing outcome measures for an intervention study and when analyzing the results of the study (psychometric properties being just one of them).

In their systematic review, Pereira and Lousada (2023) have also reported that the instruments used to measure the effects of a pragmatic intervention were mostly diagnostic instruments (e.g., CELF-4), which are typically not sensitive enough to detect subtle changes over short periods (Grzadzinski et al., 2020), as this is not their purpose either. Although many of the instruments used in the studies included in the present review were also diagnostic (e.g., Vineland-II; PLS-5; MSEL; New RDLS; Bayley-III), others were purposefully created to evaluate the progress of the social skills intervention (e.g., ASSP; BOSCC), although they did not analyze responsiveness.

It is also important to note that, as some interventions were holistic, the outcome measures used also reflected this breadth. Many studies included more outcome measures than those presented in this systematic review, but as they were not standardized, they were not considered, since the aim of this review is based on standardized instruments. However, it should be noted that some of those nonstandardized instruments could be more specific to pragmatic language skills. Thus, this systematic review also shows that many of the standardized instruments used to assess the effects of interventions are not mainly related to pragmatics (only include some items or subtests); some are diagnostic measures, and others are more general assessments considering several areas of development. This can be explained by the fact that the complex nature of pragmatics could be difficult to assess using standardized instruments. It should be highlighted that future intervention studies need to be more explicit about what characteristics of autism their interventions and outcome measures are targeting.

This review contributes to the theoretical understanding of measurement following interventions, particularly in pragmatics, for autistic children by critically examining the psychometric properties of standardized instruments used as outcome measures. While validity and reliability are essential psychometric properties, the findings pointed out a crucial gap in the existing literature: the lack of evidence regarding instrument responsiveness, which reflects an instrument's sensitivity to detecting meaningful change over time. Given that interventions aim to produce significant improvements in communication, the absence of data on responsiveness limits the ability to confidently interpret intervention outcomes. By highlighting this gap, this review advances theoretical discourse on the comprehensive assessment of psychometric properties and provides practical recommendations for future research and clinical practice. Specifically, it underscores the need for researchers to align outcome measures more precisely with the theoretical constructs and to transparently articulate the targeted abilities within the context of intervention goals. This will strengthen the validity of future findings and enhance the translation of research into effective, evidence-based practices for autistic children.

Some limitations must be acknowledged. Considering the existence of several definitions of validity, reliability, and responsiveness and the use of different measures to determine measurement properties, extracting information was challenging. Also, the extraction was particularly difficult considering that some of the instruments are very old and therefore, it is not always possible to access the information. Since the authors did not have access to the manuals of the older instruments, they may have missed some information.

Considering the results of this review, future intervention studies should better describe the rationale for the outcome measures and specify what features of autism (or co-occurring conditions) are being targeted and measured. Furthermore, it will be crucial to analyze the responsiveness of instruments used as outcome measures in future studies.

## Conclusions

This review will fill a knowledge gap in the field of interventions for children with ASD. Focusing particularly on pragmatics, it will strengthen critical thinking about the effectiveness of an intervention and the outcome measures used. Considering psychometric properties, this review emphasizes the importance of considering not only the validity and reliability of an instrument but also responsiveness to provide accurate, evidence-based decisions and interpretations. This will improve clinical decision-making around ASD.

## Supplemental Material

sj-docx-1-dli-10.1177_23969415251341251 - Supplemental material for Psychometric Properties of the Standardised Instruments that are Used to Measure (Pragmatic) Intervention Effects in Autistic Children: A Systematic Review Supplemental material, sj-docx-1-dli-10.1177_23969415251341251 for Psychometric Properties of the Standardised Instruments that are Used to Measure (Pragmatic) Intervention Effects in Autistic Children: A Systematic Review by Tatiana Pereira, Ana Cláudia Lopes, Ana Margarida Ramalho and Marisa Lousada in Autism & Developmental Language Impairments

sj-docx-2-dli-10.1177_23969415251341251 - Supplemental material for Psychometric Properties of the Standardised Instruments that are Used to Measure (Pragmatic) Intervention Effects in Autistic Children: A Systematic Review Supplemental material, sj-docx-2-dli-10.1177_23969415251341251 for Psychometric Properties of the Standardised Instruments that are Used to Measure (Pragmatic) Intervention Effects in Autistic Children: A Systematic Review by Tatiana Pereira, Ana Cláudia Lopes, Ana Margarida Ramalho and Marisa Lousada in Autism & Developmental Language Impairments

sj-docx-3-dli-10.1177_23969415251341251 - Supplemental material for Psychometric Properties of the Standardised Instruments that are Used to Measure (Pragmatic) Intervention Effects in Autistic Children: A Systematic Review Supplemental material, sj-docx-3-dli-10.1177_23969415251341251 for Psychometric Properties of the Standardised Instruments that are Used to Measure (Pragmatic) Intervention Effects in Autistic Children: A Systematic Review by Tatiana Pereira, Ana Cláudia Lopes, Ana Margarida Ramalho and Marisa Lousada in Autism & Developmental Language Impairments

sj-docx-4-dli-10.1177_23969415251341251 - Supplemental material for Psychometric Properties of the Standardised Instruments that are Used to Measure (Pragmatic) Intervention Effects in Autistic Children: A Systematic Review Supplemental material, sj-docx-4-dli-10.1177_23969415251341251 for Psychometric Properties of the Standardised Instruments that are Used to Measure (Pragmatic) Intervention Effects in Autistic Children: A Systematic Review by Tatiana Pereira, Ana Cláudia Lopes, Ana Margarida Ramalho and Marisa Lousada in Autism & Developmental Language Impairments
