# Supplementary material for: Psychometric Properties of the Standardised Instruments that are Used to Measure (Pragmatic) Intervention Effects in Autistic Children: A Systematic Review
Source: Autism Dev Lang Impair. 2025 May 7;10:23969415251341251. doi: 10.1177/23969415251341251 (PMC12078967; doi:10.1177/23969415251341251)
Supplement: sj-docx-2-dli-10.1177_23969415251341251 - Supplemental material for Psychometric Properties of the Standardised Instruments that are Used to Measure (Pragmatic) Intervention Effects in Autistic Children: A Systematic Review [file sj-docx-2-dli-10.1177_23969415251341251.docx]

| **Database** | **Papers** | **Search Strategy** | **Filters applied** |
| --- | --- | --- | --- |
| PubMed | 791 | ((‘autism spectrum disorder’ OR autism OR ASD OR autistic OR ‘autistic disorder’ OR Asperger OR ‘Asperger syndrome’ OR Rett OR ‘Rett syndrome’ OR ‘pervasive developmental disorder not otherwise specified’ OR ‘childhood disintegrative disorder’) AND (pragmatic OR ‘pragmatic language’ OR social OR ‘social communication’ OR ‘social skills’) AND (trial OR intervention OR treatment OR effect* OR efficacy OR (‘standardiz* AND instrument*) OR (outcome* AND measure*))) | Clinical trial, Randomized Controlled Trial, English, Child: birth-18years from 2005/1/1 – 2022/12/31 |
| Web of Science | 543 | TI=((‘autism spectrum disorder’ OR autism OR ASD OR autistic OR ‘autistic disorder’ OR Asperger OR ‘Asperger syndrome’ OR Rett OR ‘Rett syndrome’ OR ‘pervasive developmental disorder not otherwise specified’ OR ‘childhood disintegrative disorder’) AND (pragmatic OR ‘pragmatic language’ OR social OR ‘social communication’ OR ‘social skills’) AND (trial OR intervention OR treatment OR effect* OR efficacy OR (‘standardiz* AND instrument*) OR (outcome* AND measure*))) | Timespan: 2005-01-01 to 2022-12-31 (Publication Date) |
| CENTRAL | 1740 | ((‘autism spectrum disorder’ OR autism OR autistic OR ‘autistic disorder’ OR Asperger OR ‘Asperger syndrome’ OR Rett OR ‘Rett syndrome’ OR ‘pervasive developmental disorder not otherwise specified’ OR ‘childhood disintegrative disorder’) AND (pragmatic OR ‘pragmatic language’ OR social OR ‘social communication’ OR ‘social skills’) AND (trial OR intervention OR treatment OR effect* OR efficacy OR (‘standardiz* AND instrument*) OR (outcome* AND measure*))) | Not applied |
| Scopus | 19 | ALL('autism spectrum disorder' OR autism OR ASD OR autistic OR 'autistic disorder' OR Asperger OR 'Asperger syndrome' OR Rett OR 'Rett syndrome' OR 'pervasive developmental disorder not otherwise specified' OR 'childhood disintegrative disorder') AND (pragmatic OR 'pragmatic language' OR social OR 'social communication' OR 'social skills') AND (trial OR intervention OR treatment OR effect* OR efficacy OR ('standardiz* instrument*') OR (outcome* AND measure*)) | Language: English |
