# Supplementary material for: Psychometric Properties of the Standardised Instruments that are Used to Measure (Pragmatic) Intervention Effects in Autistic Children: A Systematic Review
Source: Autism Dev Lang Impair. 2025 May 7;10:23969415251341251. doi: 10.1177/23969415251341251 (PMC12078967; doi:10.1177/23969415251341251)
Supplement: sj-docx-3-dli-10.1177_23969415251341251 - Supplemental material for Psychometric Properties of the Standardised Instruments that are Used to Measure (Pragmatic) Intervention Effects in Autistic Children: A Systematic Review [file sj-docx-3-dli-10.1177_23969415251341251.docx]

Table S1. A synthesis of the characteristics of the included randomised controlled trials.

| **Author(s) & year** | **Study design** | **Aim(s)/research question** | **Participants** | **Intervention** | **Outcome measures*** |
| --- | --- | --- | --- | --- | --- |
| **Afsharnejad et al. (2021)** | Randomised controlled trial | Evaluating the efficacy of social skills group training KONTAKT® in comparison to a manualized interactive group cooking programme | 90 verbal autistic adolescents aged 12-17 years old with IQ > 70 | KONTAKT® is a manualised social skills group training intervention for autistic youth (8–17 years), focusing on communication and social interaction skills, social motivation, problem-solving skills, and self-confidence, aiming to support participants in achieving their personally meaningful social goals | SRS-2 |
| **Bauminger-Zviely et al. (2020)** | Randomised controlled trial | Evaluating the efficacy of School-based Peer Social Intervention (S-PSI) in minimally verbally children with ASD | 54 minimally verbal autistic children aged 8-16 years old | The S-PSI comprises two main curriculum protocols (one for conversation and one for collaboration). It aims to enhance peer interaction and social engagement in two core areas that are essential for effective social interaction but noticeably deficient in minimally verbally children with ASD namely, social collaboration and social conversation | Vineland |
| **Beaumont et al. (2021)** | Randomised controlled trial | Exploring the effectiveness of a parent-supported adaptation of the computer game-based social skills program Secret Agent Society (SAS) with an active comparison condition | 70 child-parent dyads were randomised to SAS (n = 35) or a caregiver-supported cognitive skills training game (n = 35). Child participants were on the autism spectrum (aged 7– 12) | The SAS intervention was adapted from the published SAS program targeting social and emotional skills. See Beaumont et al. (2021) | SSQ |
| **Casenhiser et al. (2013)** | Randomised controlled trial | Exploring the effectiveness of the Milton & Ethel Harris Research Initiative (MEHRI) treatment programme | 51 children with ASD (aged 2;0–4;11) | The MEHRI treatment is a DIR-based intervention aimed at improving children’s social interaction and communication abilities | PLS-4, CASL |
| **Chang et al. (2016)** | Randomised controlled trial | Examining the implementation of a modified evidence-based social communication intervention, Joint Attention Symbolic Play Engagement and Regulation (JASPER) to be adapted for delivery using small group instruction by teachers and paraprofessionals in public preschool classrooms | 66 preschool children  with ASD (aged 3–5 years) | The JASPER intervention was adapted to fit the needs of the classroom as recommended by the school staff. See Chang et al. (2016) | MSEL |
| **de Korte et al. (2021)** | Randomised controlled trial | Examining the efficacy of clinician delivered Pivotal Response Treatment (PRT) in school-aged children and adolescents with ASD, integrating parent training and involvement of teachers | 44 children with ASD (aged 9–15 years) | The treatment focused on creating different social communication learning opportunities for the child and on training parents/teachers to implement PRT principles (e.g., child’s choice, interspersing maintenance and acquisition tasks, task variation, natural and direct rewards, and rewarding attempts) in child’s natural environment | SRS, ADOS-2, Vineland-II |
| **DeRosier et al. (2011)** | Randomised controlled trial | Investigating the efficacy of the Social Skills Group Intervention-High Functioning Autism (S.S.GRIN-HFA) | 55 children with a prior diagnosis of ASD (aged 8–12 years) and 55 parents (i.e., one parent per child participant) | The S.S.GRIN-HFA is a 15-week, group social skills training intervention designed to improve the social skills and social relationships of 8- to 12-year-old children diagnosed with high functioning ASD | SRS |
| **Dolan et al. (2016)** | Randomised controlled trial | Examining the effectiveness of Program for the Education and Enrichment of Relational Skills (PEERS®) for improving social skills among adolescents with ASD | 58 participants with ASD (aged 11–16 years old) | The PEERS® is a manualized treatment program designed to teach motivated adolescents with high functioning ASD the social skills needed to make and keep friends | TASSK |
| **Fletcher-Watson et al. (2016)** | Randomised controlled trial | Evaluating the impact of the FindMe (iPad application) in young children with ASD | 54 children (aged under 6 years) with a diagnosis of ASD | The FindMe aimed to enhance the real-world social communication skills of the children through motivating, daily rehearsal of very basic sub-skills. The app was designed to give children an opportunity to rehearse two key social communication skills: attending to people and following social cues | CSBS, ADOS-2 |
| **Frankel et al. (2010)** | Randomised Controlled Trial | Evaluating the effectiveness of Children’s Friendship Training (CFT), a manualized parent-assisted intervention to improve social skills among second to fifth grade children with ASD. Comparison was made with a delayed treatment control group | 68 children with ASD attending a 2^nd^ through 5^th^ grade regular classroom | The CFT intervention treatment was composed of 12 weekly sessions of 60 min in length. Targeted skills included conversational skills, peer entry skills,  developing friendship networks, good sportsmanship, good host behaviour during play dates, and handling teasing | SSRS |
| **Gengoux et al. (2019)** | Randomised controlled trial | Evaluating the effectiveness of a Pivotal Response Treatment package (PRT-P) consisting of parent training and clinician-delivered in-home intervention | 48 children with ASD and significant language delay  (aged 2–5 years) | The PRT-P treatment consisted of two phases: an intensive phase and a maintenance phase. The intensive phase is from week 1 to week 12, during which parents received weekly 60-minute training sessions and children received 10 hours per week of clinician delivered in-home treatment). The maintenance phase is from week 12 to week 24, during which parents received monthly 60-minute training sessions and children received 5 hours per week of in-home treatment) | Vineland-II, MSEL, SRS-2) |
| **Hopkins et al. (2011)** | Randomised controlled trial | Assessing the efficacy of FaceSay, a computer-based social skills training program for children with ASD |  | The FaceSay is a colourful program that contains three different games with realistic avatars designed to teach children specific social skills. The overall goal of the games is to promote awareness of the movements and features of the face, particularly the area around the eyes | SSRS |
| **Ingersoll (2012)** | Randomised controlled trial | Evaluating the effects of a focused imitation intervention on social functioning (initiation of joint attention and social-emotional skills) in children with ASD | 29 children with ASD (aged 27–47 months) | Reciprocal Imitation Training (RIT) uses a blend of naturalistic behavioural and developmental strategies to teach imitation within a social-interactive context | Bayley-III |
| **Kasari et al. (2014)** | Randomised controlled trial | Comparing the efficacy of two short-term, low-intensity, caregiver-mediated intervention interventions (Caregiver-Mediated Module (CMM) or Caregiver Education Module (CEM)) for preschool-aged children with ASD who had low resources | 112 children with ASD (aged 2–5 years) and their caregivers | The CMM group involved 21-hour sessions per week for 12 weeks in the home. Caregivers were actively coached in the treatment model with their child by  trained interventionists. The CEM group involved small group-based caregiver training without the child being present. Caregivers gathered in neighbourhood locations, such as homes, community centers, clinics, and schools. The caregivers attended 2-hour group sessions each week that covered similar material to the CMM intervention, with a focus on teaching communication to their children, the ABCs of behaviour management, and developing routines | ADOS, MSEL |
| **LaGasse (2014)** | Randomised controlled trial | Examining the effects of a Music Therapy Group (MTG) intervention on eye gaze, joint attention, and communication in children with ASD | 17 children (aged 6–9), with ASD were randomly assigned to MTG or the no-music Social Skills Group (SSG) | In the MTG, the transformational design model was used to create music experiences that were functionally like the non-musical experiences, with the addition of music and cues to facilitate the desired social skills. The SSG included cooperative play experiences that involved taking turns, passing cards/game pieces, and interacting with their peers. Each group met for 50-minutes, twice a week, for 5 weeks | SRS |
| **Landa et al. (2011)** | Randomised controlled trial | Evaluating the impact of supplementing a comprehensive intervention with a curriculum targeting socially synchronous behavior on social outcomes of toddlers with ASD | 50 toddlers with ASD (aged 21–33 months) | The curriculum used within both conditions was the Assessment, Evaluation, and Programming System for Infants and Children (AEPS), a comprehensive developmental curriculum that provides guidelines for complexity of intervention goals based on children’s individual developmental profile. Intervention was provided within a Kennedy Krieger classroom four days per week for 2.5 hours per day for six months | CSBS, MSEL |
| **Leaf et al. (2017)** | Randomised controlled trial | Evaluating the effects of a behaviourally based Social Skills Group (SSG) | 15 children under 6 years old with ASD | See Leaf et al. (2017) | SSRS, SRS |
| **Lopata et al. (2010)** | Randomised controlled trial | Examining the efficacy of a manualized social intervention for children diagnosed with high-functioning ASD | 36 children (aged 7–12 years) diagnosed with high-functioning ASD | See Lopata et al. (2010) | SRS, CASL |
| **Lopata et al. (2016)** | Randomised controlled trial | Evaluating the efficacy of Mind Reading (MR) as a component of a comprehensive psychosocial treatment | 36 children (aged 7–12 years) diagnosed with high-functioning ASD | All participants received the comprehensive 5-week summer treatment (summerMAX), with half randomly assigned to also receive MR (emotion recognition) computer instruction as part of the comprehensive treatment (summerMAX + MR) | SRS |
| **Lopata et al. (2021)** | Randomised controlled trial | Evaluating the efficacy of the outpatient model (MAXout) for children with ASD without intellectual disability | 88 children (aged 7–12 years) with ASD and without intellectual disability | See Lopata et al. (2021) | SRS-2, CASL |
| **Mohammadzaheri et al. (2021)** | Randomised controlled trial | Examining the effects of Pivotal Response Treatment (PRT) to improve verbal initiations in children with ASD | 20 children with ASD (aged 6–12 years) | See Mohammadzaheri et al. (2021) | CCC |
| **Murphy et al. (2014)** | Randomised controlled trial | Evaluating an intervention to support children with social communication difficulties to participate more effectively in peer collaborative work | 32 children (aged 5–6 years) with social communication difficulties (including children with ASD and other conditions) | Children received an intervention based on a dyadic computer task (Maze Task) requiring substantial high-level collaboration and perspective-taking. The Maze Task aims to highlight that different people may have different visual perspectives | TPS |
| **Olsson et al. (2017)** | Randomised controlled trial | Evaluating the effectiveness of the Social Skills Group Training (SSGT) KONTAKT® delivered by regular clinical staff, as a complement to standard care | Participants (n = 296; 88 females and 208 males) were children (n = 172) and adolescents (n = 124) aged 8–17 years with ASD without intellectual disability | KONTAKT® is a manualised social skills group training intervention to improve social interaction skills, social motivation, problem-solving skills, and self-confidence, aiming to support participants in achieving their personally meaningful social goals | SRS |
| **Płatos et al. (2023)** | Randomised controlled trial | Examining the efficacy of the Polish adaptation of the PEERS® curriculum for adolescents on the autism spectrum | 29 adolescents (aged 11–16) with ASD | PEERS® for Adolescents is a parent-assisted social skills training that uses cognitive-behavioral techniques to improve friendship quality and social skills among adolescents with ASD | TASSK-R, SRS-2 |
| **Rabin et al. (2018)** | Randomised controlled trial | Evaluating the effectiveness of the adapted and translated Hebrew version of the PEERS® intervention | 41 adolescents with ASD and no intellectual disability (aged 12–17 years) | PEERS®. See Rabin et al. (2018) | TASSK, SSIS, SRS-2 |
| **Rice et al. (2015)** | Randomised controlled trial | Determining the effects of the FaceSay computer program on the ability of children with ASD to recognize emotions, understand another’s perspective, and improve their social skills in comparison to other ASD children not receiving the intervention | 31 elementary school students in Ventura County, California, aged 5–11 years (M = 7.77) | FaceSay. See Rice et al. (2015) | SRS-2 |
| **Roberts et al. (2011)** | Randomised controlled trial | Evaluating the effectiveness of early intervention delivered to preschool-age children with ASD (Building Blocks program) by comparing two variations of the program: a home-based service and a centre-based small group program. A wait list control group was also recruited | 84 preschool-age children with ASD | Building Blocks. See Roberts et al. (2011) | Vineland-II, RDLS |
| **Roberts et al. (2023)** | Randomised controlled trial | Examining the effects of a hybrid intervention (JASP-EMT) that blended two interventions: Enhanced Milieu Teaching (EMT) and Joint Attention, Symbolic Play, Engagement and Regulation (JASPER) | 120 caregivers and their autistic children (aged 24–36 months) | The JASP-EMT builds on the JASPER intervention to teach the social foundations of communication and the EMT intervention to teach spoken language. Children in the intervention group received JASP-EMT and children in the control group received positive behaviour support | CSBS, BOSCC, PLS-5 |
| **Rogers et al. (2019)** | Randomised controlled trial | Testing the effects of an enhanced low-intensity version of the parent-implemented Early Start Denver Model (P-ESDM) | 45 children with ASD (aged 12–30 months) | In this study, two versions of the P-ESDM were used: the basic model, in which we delivered 1.5h of clinic-based parent coaching weekly, and an enhanced version that contained three additions: motivational interviewing, multimodal learning tools, and a weekly 1.5h home visit. The interventions were delivered for 12 weeks | ADOS - Toddler Module, MSEL, Vineland-II |
| **Rollins and De Froy (2023)** | Randomised controlled trial | Examining the efficacy of Pathways Early Autism Intervention | 69 caregivers and children aged 18–50 months | See Rollins and De Froy (2023) | CSBS |
| **Schertz et al. (2013)** | Randomised controlled trial | Determining the effects of the Joint Attention Mediated Learning (JAML) intervention on acquisition of joint attention and other early social communication competencies for toddlers with ASD | 23 toddlers with ASD under age 30 months | Joint Attention Mediated Learning (JAML). See Schertz et al. (2013) | MSEL, Vineland-II |
| **Schohl et al. (2014)** | Randomised controlled trial | Evaluating the effects of the Program for the Education and Enrichment of Relational Skills (PEERS) | 58 participants aged 11–16 years | The PEERS® focuses on improving friendship quality and social skills among adolescents with ASD | TASSK, Vineland-II, ADOS-Generic, SSRS, SRS |
| **Schwartzberg and Silverman (2013)** | Randomised controlled trial | Examining the effects of music-based social stories on the comprehension and generalization of social skills in children with ASD | 30 participants (aged 9–21 years; M = 15.78 and SD = 3.27; 29 male and 1 female) with ASD | Music-based social stories. See Schwartzberg and Silverman (2013) | ASSP |
| **Shum et al. (2019)** | Randomised controlled trial | Examining the treatment efficacy of the PEERS® among Chinese adolescents with ASD | 72 adolescents (aged 11–15 years; M = 13.51, SD = 0.97; 57 males and 15 females) studying at local secondary schools in Hong Kong and their parents | PEERS®. See Shum et al. (2019). | ABAS-II, SRS-2, TASSK |
| **Siller and Sigman (2008)** | Randomised controlled trial | Evaluating the efficacy of the Focused Playtime Intervention (FPI) | 70 children (64 males and 6 females) with diagnostic criteria for autistic disorder | The FPI aims to enhance responsive parental behaviours in the context of parent-child play interactions | MSEL |
| **Soorya et al. (2015)** | Randomised controlled trial | Evaluating the efficacy of a targeted social skills training group (Seaver-NETT: Non-verbal communication, Emotion recognition, and Theory of mind Training) in school-aged children with ASD | 69 children with ASD (aged 8–11 years) with verbal IQ greater than 70 | The intervention, Seaver-NETT is a 12-session cognitive-behavioral intervention for verbal, school-aged children | SRS, CCC-2 |
| **Thomeer et al. (2015)** | Randomised controlled trial | Evaluating the efficacy of a computer software - Mind Reading (MR) - and in vivo rehearsal treatment on the emotion decoding and encoding skills, autism symptoms, and social skills | 43 children (aged 7–12 years) with high-functioning ASD | The MR is an interactive software program designed to teach recognition of simple and complex emotions to children with ASD via facial-video and vocal-audio stimuli. The program consists of 412 emotions, organized into 24 emotion groups and by 6 emotion levels | SRS |
| **Thomeer et al. (2019)** | Randomised controlled trial | Examiming the feasibility and effectiveness of a comprehensive psychosocial treatment, summerMAX, when implemented by a community agency | 57 high-functioning children (48 male, 9 female), aged 7–12 years with ASD | The 5-week summerMAX treatment included instruction and therapeutic activities targeting social/social-communication skills, interpretation of nonliteral language skills, face-emotion recognition skills, and interest expansion. A behavioural program was also used to increase skills acquisition and decrease autism spectrum disorder symptoms and problem behaviours | CASL, SRS-2 |
| **Valeri et al. (2020)** | Randomised controlled trial | Evaluating the potential benefit of Cooperative Parent-Mediated Therapy (CPMT) | 34 participants with ASD (7 females; 27 males; aged 2, 6, 11 years) and their parents | The CPMT aimed to improve parental skills, to enable parents to promote in their child the following seven target skills: socio-emotional engagement, emotional regulation, imitation, communication, joint attention, play and cognitive flexibility, and cooperative interaction | ADOS Calibrated Severity Scores for ADOS-Generic |
| **van den Berk-Smeekens et al. (2021)** | Randomised controlled trial | Examining the efficacy of Pivotal Response Treatment (PRT) with and without robot-assistance, compared to Treatment As Usual (TAU) | 73 young children (PRT: n = 25; PRT + robot: n = 25; TAU: n = 23) with ASD (aged 3–8 years) | See van den Berk-Smeekens et al. (2021) | SRS, ADOS-2 |
| **Vernon et al. (2018)** | Randomised controlled trial | Evaluating the impact of the 20-week Social Tools And Rules for Teens (START) Program on the social functioning of adolescents with ASD | 40 adolescents (aged 12–17) with a diagnosis of ASD | START. See Vernon et al. (2018) | SSIS, SRS-2 |
| **Williams et al. (2012)** | Randomised controlled trial | Evaluating the efficacy of an emotion training programme for a group of young children with ASD with a range of intellectual ability | 55 children with ASD (aged 4–7 years). Children were randomly assigned to an intervention (n = 28) or control group (n = 27) | See Williams et al. (2012) | Vineland-II, ADOS |
| **Yoo et al. (2014)** | Randomised controlled trial | Examining the feasibility and treatment efficacy of a Korean version of PEERS® for enhancing social skills in adolescents with ASD | 47 adolescents (aged 12–18 years) with ASD and a verbal IQ ≥ 65 | The English version of the PEERS® Treatment Manual was translated into Korean and reviewed. See Yoo et al. (2014) | ADOS, Vineland, TASSK-R, SSRS, SRS |

*Considering the aim of this review, only standardized outcome measures used will be listed. Legend: IQ - intelligence quotient; ASD - autism spectrum disorder; M - mean; SD - standard deviation; SRS - Social Responsiveness Scale; Vineland - Vineland Adaptative Behaviour Scale; SSQ – Social Skills Questionnaire; PLS - Preschool Language Scale; CASL - Comprehensive Assessment of Spoken Language; MSEL - Mullen Scales of Early Learning; ADOS - Autism Diagnostic Observation Scale; TASSK - Test of Adolescent Social Skills Knowledge; CSBS - Communication and Symbolic Behaviour Scale; SSRS - Social Skills Rating System; Bayley - Bayley Scales of Infant Development; CCC - Children’s Communication Checklist; TPS - Test of Pragmatic Skills; ASSP - Autism Social Skills Profile; SSIS - Social Skills Improvement System; STAT - Screening Tool for Autism in Toddlers and Young Children; ABAS - Adaptive Behavior Assessment System.

**References**

Afsharnejad, B., Falkmer, M., Black, M. H., Alach, T., Lenhard, F., Fridell, A., Coco, C., Milne, K., Bolte, S., & Girdler, S. (2021). KONTAKT social skills group training for Australian adolescents with autism spectrum disorder: a Randomised controlled trial. *European child & adolescent psychiatry*. <https://doi.org/10.1007/s00787-021-01814-6>

Bauminger-Zviely, N., Eytan, D., Hoshmand, S., & Rajwan Ben-Shlomo, O. (2020). Preschool Peer Social Intervention (PPSI) to Enhance Social Play, Interaction, and Conversation: study Outcomes [Journal: Article]. *Journal of Autism and Developmental Disorders*, *50*(3), 844‐863. <https://doi.org/10.1007/s10803-019-04316-2>

Beaumont, R., Walker, H., Weiss, J., & Sofronoff, K. (2021). Randomised Controlled Trial of a Video Gaming-Based Social Skills Program for Children on the Autism Spectrum [Journal Article; Randomised Controlled Trial]. *Journal of Autism and Developmental Disorders*, *51*(10), 3637‐3650. <https://doi.org/10.1007/s10803-020-04801-z>

Casenhiser, D. M., Shanker, S. G., & Stieben, J. (2013). Learning through interaction in children with autism: preliminary data from asocial-communication-based intervention [Journal Article; Randomised Controlled Trial; Research Support, Non‐U.S. Gov't]. *Autism*, *17*(2), 220‐241. <https://doi.org/10.1177/1362361311422052>

Chang, Y. C., Shire, S. Y., Shih, W., Gelfand, C., & Kasari, C. (2016). Preschool Deployment of Evidence-Based Social Communication Intervention: JASPER in the Classroom [Journal: Article]. *Journal of Autism and Developmental Disorders*, *46*(6), 2211‐2223. <https://doi.org/10.1007/s10803-016-2752-2>

de Korte, M. W. P., van den Berk-Smeekens, I., Buitelaar, J. K., Staal, W. G., & van Dongen-Boomsma, M. (2021). Pivotal Response Treatment for School-Aged Children and Adolescents with Autism Spectrum Disorder: a Randomised Controlled Trial [Journal Article; Randomised Controlled Trial]. *Journal of Autism and Developmental Disorders*, *51*(12), 4506‐4519. <https://doi.org/10.1007/s10803-021-04886-0>

DeRosier, M. E., Swick, D. C., Davis, N. O., McMillen, J. S., & Matthews, R. (2011). The efficacy of a Social Skills Group Intervention for improving social behaviors in children with High Functioning Autism Spectrum disorders [Journal Article; Randomised Controlled Trial; Research Support, N.I.H., Extramural]. *Journal of Autism and Developmental Disorders*, *41*(8), 1033‐1043. <https://doi.org/10.1007/s10803-010-1128-2>

Dolan, B. K., Van Hecke, A. V., Carson, A. M., Karst, J. S., Stevens, S., Schohl, K. A., Potts, S., Kahne, J., Linneman, N., Remmel, R., & et al. (2016). Brief Report: assessment of Intervention Effects on In Vivo Peer Interactions in Adolescents with Autism Spectrum Disorder (ASD) [Journal Article; Randomised Controlled Trial; Research Support, Non‐U.S. Gov't]. *Journal of Autism and Developmental Disorders*, *46*(6), 2251‐2259. <https://doi.org/10.1007/s10803-016-2738-0>

Fletcher-Watson, S., Petrou, A., Scott-Barrett, J., Dicks, P., Graham, C., O’Hare, A., Pain, H., & McConachie, H. (2016). A trial of an iPad™ intervention targeting social communication skills in children with autism [Academic Journal]. *Autism: the international journal of research & practice*, *20*(7), 771‐782. <https://doi.org/10.1177/1362361315605624>

Frankel, F., Myatt, R., Sugar, C., Whitham, C., Gorospe, C. M., & Laugeson, E. (2010). A Randomised controlled study of parent-assisted Children's Friendship Training with children having autism spectrum disorders [Journal Article; Randomised Controlled Trial; Research Support, N.I.H., Extramural]. *Journal of Autism and Developmental Disorders*, *40*(7), 827‐842. <https://doi.org/10.1007/s10803-009-0932-z>

Gengoux, G. W., Abrams, D. A., Schuck, R., Millan, M. E., Libove, R., Ardel, C. M., Phillips, J. M., Fox, M., Frazier, T. W., & Hardan, A. Y. (2019). A Pivotal Response Treatment Package for Children With Autism Spectrum Disorder: an RCT [Journal Article; Randomised Controlled Trial; Research Support, N.I.H., Extramural]. *Pediatrics*, *144*(3). <https://doi.org/10.1542/peds.2019-0178>

Hopkins, I. M., Gower, M. W., Perez, T. A., Smith, D. S., Amthor, F. R., Wimsatt, F. C., & Biasini, F. J. (2011). Avatar assistant: improving social skills in students with an ASD through a computer-based intervention [Journal Article; Randomised Controlled Trial; Research Support, Non‐U.S. Gov't]. *Journal of Autism and Developmental Disorders*, *41*(11), 1543‐1555. <https://doi.org/10.1007/s10803-011-1179-z>

Ingersoll, B. (2012). Brief report: effect of a focused imitation intervention on social functioning in children with autism [Journal Article; Randomised Controlled Trial]. *Journal of Autism and Developmental Disorders*, *42*(8), 1768‐1773. <https://doi.org/10.1007/s10803-011-1423-6>

Kasari, C., Lawton, K., Shih, W., Barker, T. V., Landa, R., Lord, C., Orlich, F., King, B., Wetherby, A., & Senturk, D. (2014). Caregiver-mediated intervention for low-resourced preschoolers with autism: an RCT [Journal Article; Randomised Controlled Trial; Research Support, N.I.H., Extramural]. *Pediatrics*, *134*(1), e72‐79. <https://doi.org/10.1542/peds.2013-3229>

LaGasse, A. B. (2014). Effects of a music therapy group intervention on enhancing social skills in children with autism [Journal Article; Randomised Controlled Trial; Research Support, Non‐U.S. Gov't]. *Journal of music therapy*, *51*(3), 250‐275. <https://doi.org/10.1093/jmt/thu012>

Landa, R. J., Holman, K. C., O'Neill, A. H., & Stuart, E. A. (2011). Intervention targeting development of socially synchronous engagement in toddlers with autism spectrum disorder: a Randomised controlled trial [Journal Article; Randomised Controlled Trial]. *Journal of child psychology and psychiatry, and allied disciplines*, *52*(1), 13‐21. <https://doi.org/10.1111/j.1469-7610.2010.02288.x>

Leaf, J. B., Leaf, J. A., Milne, C., Taubman, M., Oppenheim-Leaf, M., Torres, N., Townley-Cochran, D., Leaf, R., McEachin, J., & Yoder, P. (2017). An Evaluation of a Behaviorally Based Social Skills Group for Individuals Diagnosed with Autism Spectrum Disorder [Journal Article; Randomised Controlled Trial]. *Journal of Autism and Developmental Disorders*, *47*(2), 243‐259. <https://doi.org/10.1007/s10803-016-2949-4>

Lopata, C., Thomeer, M. L., Rodgers, J. D., Donnelly, J. P., & Booth, A. J. (2021). RCT of a Comprehensive Outpatient Treatment for Children with Autism Spectrum Disorder [Journal Article; Randomised Controlled Trial; Research Support, U.S. Gov't, Non‐P.H.S.]. *Journal of clinical child and adolescent psychology*, *50*(6), 796‐810. <https://doi.org/10.1080/15374416.2020.1790380>

Lopata, C., Thomeer, M. L., Rodgers, J. D., Donnelly, J. P., & McDonald, C. A. (2016). RCT of mind reading as a component of a psychosocial treatment for high-functioning children with ASD [Journal: Article]. *Research in Autism Spectrum Disorders*, *21*, 25‐36. <https://doi.org/10.1016/j.rasd.2015.09.003>

Lopata, C., Thomeer, M. L., Volker, M. A., Toomey, J. A., Nida, R. E., Lee, G. K., Smerbeck, A. M., & Rodgers, J. D. (2010). RCT of a manualized social treatment for high-functioning autism spectrum disorders [Journal Article; Randomised Controlled Trial]. *Journal of Autism and Developmental Disorders*, *40*(11), 1297‐1310. <https://doi.org/10.1007/s10803-010-0989-8>

Mohammadzaheri, F., Koegel, L. K., Bakhshi, E., Khosrowabadi, R., & Soleymani, Z. (2021). The Effect of Teaching Initiations on the Communication of Children with Autism Spectrum Disorder: a Randomised Clinical Trial [Journal: Article in Press]. *Journal of Autism and Developmental Disorders*. <https://doi.org/10.1007/s10803-021-05153-y>

Murphy, S. M., Faulkner, D. M., & Reynolds, L. R. (2014). A randomised controlled trial of a computerised intervention for children with social communication difficulties to support peer collaboration [Article]. *Research in Developmental Disabilities*, *35*(11), 2821-2839. <https://doi.org/10.1016/j.ridd.2014.07.026>

Olsson, N. C., Flygare, O., Coco, C., Gorling, A., Rade, A., Chen, Q., Lindstedt, K., Berggren, S., Serlachius, E., Jonsson, U., Tammimies, K., KjeIlin, L., & Bolte, S. (2017). Social Skills Training for Children and Adolescents With Autism Spectrum Disorder: A Randomised Controlled Trial [Article]. *Journal of the American Academy of Child and Adolescent Psychiatry*, *56*(7), 585-592. <https://doi.org/10.1016/j.jaac.2017.05.001>

Płatos, M., Wojaczek, K., & Laugeson, E. A. (2023). Effects of Social Skills Training for Adolescents on the Autism Spectrum: a Randomised Controlled Trial of the Polish Adaptation of the PEERS® Intervention via Hybrid and In-Person Delivery. *Journal of Autism and Developmental Disorders*, *53*, 4132-4146. <https://doi.org/https://doi.org/10.1007/s10803-022-05714-9>

Rabin, S. J., Israel-Yaacov, S., Laugeson, E. A., Mor-Snir, I., & Golan, O. (2018). A Randomised controlled trial evaluating the Hebrew adaptation of the PEERS® intervention: behavioral and questionnaire-based outcomes [Journal Article; Randomised Controlled Trial; Research Support, Non‐U.S. Gov't]. *Autism Research*, *11*(8), 1187‐1200. <https://doi.org/10.1002/aur.1974>

Rice, L. M., Wall, C. A., Fogel, A., & Shic, F. (2015). Computer-Assisted Face Processing Instruction Improves Emotion Recognition, Mentalizing, and Social Skills in Students with ASD [Journal Article; Randomised Controlled Trial]. *Journal of Autism and Developmental Disorders*, *45*(7), 2176‐2186. <https://doi.org/10.1007/s10803-015-2380-2>

Roberts, J., Williams, K., Carter, M., Evans, D., Parmenter, T., Silove, N., Clark, T., & Warren, A. (2011). A randomised controlled trial of two early intervention programs for young children with autism: centre-based with parent program and home-based [Journal: Article]. *Research in Autism Spectrum Disorders*, *5*(4), 1553‐1566. <https://doi.org/10.1016/j.rasd.2011.03.001>

Roberts, M. Y., Stern, Y. S., Grauzer, J., Nietfeld, J., Thompson, S., Jones, M., Kaat, A. J., & Kaiser, A. P. (2023). Teaching Caregivers to Support Social Communication: Results From a Randomised Clinical Trial of Autistic Toddlers. *American journal of speech-language pathology*, *32*, 115-127. <https://doi.org/https://doi.org/10.1044/2022_AJSLP-22-00133>

Rogers, S. J., Estes, A., Vismara, L., Munson, J., Zierhut, C., Greenson, J., Dawson, G., Rocha, M., Sugar, C., Senturk, D., & et al. (2019). Enhancing Low-Intensity Coaching in Parent Implemented Early Start Denver Model Intervention for Early Autism: a Randomised Comparison Treatment Trial [Comparative Study; Journal Article; Randomised Controlled Trial]. *Journal of Autism and Developmental Disorders*, *49*(2), 632‐646. <https://doi.org/10.1007/s10803-018-3740-5>

Rollins, P. R., & De Froy, A. M. (2023). Reexamining Pathways Early Autism Intervention in Children Before and After the Third Birthday: A Randomised Control Trial. *Journal of Autism and Developmental Disorders*, *53*, 1189-1201. <https://doi.org/https://doi.org/10.1007/s10803-022-05599-8>

Schertz, H. H., Odom, S. L., Baggett, K. M., & Sideris, J. H. (2013). Effects of Joint Attention Mediated Learning for toddlers with autism spectrum disorders: An initial Randomised controlled study. *Early Childhood Research Quarterly*, *28*, 249-258. <https://doi.org/http://dx.doi.org/10.1016/j.ecresq.2012.06.006>

Schohl, K. A., Van Hecke, A. V., Carson, A. M., Dolan, B., Karst, J., & Stevens, S. (2014). A replication and extension of the PEERS intervention: examining effects on social skills and social anxiety in adolescents with autism spectrum disorders [Journal Article; Randomised Controlled Trial; Research Support, Non‐U.S. Gov't]. *Journal of Autism and Developmental Disorders*, *44*(3), 532‐545. <https://doi.org/10.1007/s10803-013-1900-1>

Schwartzberg, E. T., & Silverman, M. J. (2013). Effects of music-based social stories on comprehension and generalization of social skills in children with autism spectrum disorders: a Randomised effectiveness study [Journal: Article]. *Arts in psychotherapy*, *40*(3), 331‐337. <https://doi.org/10.1016/j.aip.2013.06.001>

Shum, K.-M., Cho, W. K., Lam, L. M. O., Laugeson, E. A., Wong, W. S., & Law, L. S. K. (2019). Learning How to Make Friends for Chinese Adolescents with Autism Spectrum Disorder: A Randomised Controlled Trial of the Hong Kong Chinese Version of the PEERS® Intervention [Journal: Article]. *Journal of Autism and Developmental Disorders*, *49*(2), 527‐541. <https://doi.org/10.1007/s10803-018-3728-1>

Siller, M., & Sigman, M. (2008). Modeling Longitudinal Change in the Language Abilities of Children With Autism: Parent Behaviors and Child Characteristics as Predictors of Change [Article; Proceedings Paper]. *Developmental Psychology*, *44*(6), 1691-1704. <https://doi.org/10.1037/a0013771>

Soorya, L. V., Siper, P. M., Beck, T., Soffes, S., Halpern, D., Gorenstein, M., Kolevzon, A., Buxbaum, J., & Wang, A. T. (2015). Randomised comparative trial of a social cognitive skills group for children with autism spectrum disorder [Comparative Study; Journal Article; Randomised Controlled Trial; Research Support, N.I.H., Extramural; Research Support, Non‐U.S. Gov't]. *Journal of the American Academy of Child and Adolescent Psychiatry*, *54*(3), 208‐216.e201. <https://doi.org/10.1016/j.jaac.2014.12.005>

Thomeer, M. L., Lopata, C., Donnelly, J. P., Booth, A., Shanahan, A., Federiconi, V., McDonald, C. A., & Rodgers, J. D. (2019). Community Effectiveness RCT of a Comprehensive Psychosocial Treatment for High-Functioning Children With ASD [Journal Article; Randomised Controlled Trial; Research Support, Non‐U.S. Gov't]. *Journal of clinical child and adolescent psychology*, *48*(sup1), S119‐S130. <https://doi.org/10.1080/15374416.2016.1247359>

Thomeer, M. L., Smith, R. A., Lopata, C., Volker, M. A., Lipinski, A. M., Rodgers, J. D., McDonald, C. A., & Lee, G. K. (2015). Randomised Controlled Trial of Mind Reading and In Vivo Rehearsal for High-Functioning Children with ASD [Journal Article; Randomised Controlled Trial; Research Support, Non‐U.S. Gov't]. *Journal of Autism and Developmental Disorders*, *45*(7), 2115‐2127. <https://doi.org/10.1007/s10803-015-2374-0>

Valeri, G., Casula, L., Menghini, D., Amendola, F. A., Napoli, E., Pasqualetti, P., & Vicari, S. (2020). Cooperative parent-mediated therapy for Italian preschool children with autism spectrum disorder: a Randomised controlled trial [Journal Article; Randomised Controlled Trial]. *European child & adolescent psychiatry*, *29*(7), 935‐946. <https://doi.org/10.1007/s00787-019-01395-5>

van den Berk-Smeekens, I., de Korte, M. W. P., van Dongen-Boomsma, M., Oosterling, I. J., den Boer, J. C., Barakova, E. I., Lourens, T., Glennon, J. C., Staal, W. G., & Buitelaar, J. K. (2021). Pivotal Response Treatment with and without robot-assistance for children with autism: a Randomised controlled trial [Journal: Article in Press]. *European child & adolescent psychiatry*. <https://doi.org/10.1007/s00787-021-01804-8>

Vernon, T. W., Miller, A. R., Ko, J. A., Barrett, A. C., & McGarry, E. S. (2018). A Randomised Controlled Trial of the Social Tools And Rules for Teens (START) Program: an Immersive Socialization Intervention for Adolescents with Autism Spectrum Disorder [Academic Journal]. *Journal of Autism and Developmental Disorders*, *48*(3), 892‐904. <https://doi.org/10.1007/s10803-017-3380-1>

Williams, B. T., Gray, K. M., & Tonge, B. J. (2012). Teaching emotion recognition skills to young children with autism: a randomised controlled trial of an emotion training programme [Journal Article; Randomised Controlled Trial; Research Support, Non‐U.S. Gov't]. *Journal of child psychology and psychiatry, and allied disciplines*, *53*(12), 1268‐1276. <https://doi.org/10.1111/j.1469-7610.2012.02593.x>

Yoo, H. J., Bahn, G., Cho, I. H., Kim, E. K., Kim, J. H., Min, J. W., Lee, W. H., Seo, J. S., Jun, S. S., Bong, G., & et al. (2014). A Randomised controlled trial of the Korean version of the PEERS(®) parent-assisted social skills training program for teens with ASD [Comparative Study; Journal Article; Multicenter Study; Randomised Controlled Trial; Research Support, Non‐U.S. Gov't; Validation Study]. *Autism Research*, *7*(1), 145‐161. <https://doi.org/10.1002/aur.1354>
