# Supplementary material for: Psychometric Properties of the Standardised Instruments that are Used to Measure (Pragmatic) Intervention Effects in Autistic Children: A Systematic Review
Source: Autism Dev Lang Impair. 2025 May 7;10:23969415251341251. doi: 10.1177/23969415251341251 (PMC12078967; doi:10.1177/23969415251341251)
Supplement: sj-docx-4-dli-10.1177_23969415251341251 - Supplemental material for Psychometric Properties of the Standardised Instruments that are Used to Measure (Pragmatic) Intervention Effects in Autistic Children: A Systematic Review [file sj-docx-4-dli-10.1177_23969415251341251.docx]

Table S2. A synthesis of the characteristics of the included non-randomised controlled trials.

| **Author(s) & year** | **Study design** | **Aim(s)/research question** | **Participants** | **Intervention** | **Outcome measures*** |
| --- | --- | --- | --- | --- | --- |
| **Chester et al. (2019)** | Non-randomised controlled trial | Evaluating the effectiveness of an 8-week Social Skills Training (SST) intervention with a play component (unstructured versus  semi-structured) for children with ASD | 45 children with ASD aged 8–12 years (M = 10.16, SD = 1.26) | See Chester et al. (2019) | SSIS |
| **Laugeson et al. (2014)** | Non-randomised controlled trial | Examining the effectiveness of the Program for the Education and Enrichment of Relational Skills (PEERS) Curriculum for School-Based Professionals compared to daily instruction using another manualized social skills curriculum | 73 adolescents between 12 and 14 years of age with a previous diagnosis of ASD without intellectual disability, along with their parents and teachers | The PEERS Curriculum for School-Based Professionals is a manualized, school-based, teacher-facilitated social skills intervention, adapted from an evidence-based social skills program for adolescents with a diagnosis of ASD. It consisted of daily 30-min lessons, delivered 5 days per week over the course of a 14-week semester term | SRS, SSRS, TASSK |
| **Pereira et al. (2022)** | Non-randomised controlled trial | Evaluating the effects of the Pragmatic Intervention Programme (PICP) in preschool-age children with pragmatic language impairments, compared to no treatment | 20 preschool-age children with pragmatic impairment arising from both Autism Spectrum Disorder (ASD) and Developmental Language Disorder (DLD) | The PICP is a manualized intervention programme that aims to promote several skills (e.g., joint attention, turn-taking, communicative response, communicative initiative). Each child received 24 PICP-based intervention sessions, biweekly, for one our each, in preschool. Several communicative partners were involved during the sessions | TL-ALPE, EAC |
| **Ratcliffe et al. (2014)** | Non-randomised controlled trial | Evaluating the effectiveness of delivering Emotion-Based Social Skills Training (EBSST) to students, their parents, and teachers by school counsellors in schools | 217 children (aged 7–13 years) with ASD without intellectual disability attending mainstream primary schools in Australia | The EBSST aims to enhance emotional competence in primary school aged children with ASD. EBSST draws on theories of emotional development and emotional intelligence to teach children with ASD how to understand their own and other’s emotions, emotional problem solving and emotional regulation skills | SSIS |
| **Vivanti et al. (2014)** | Non-randomised controlled trial | Analysing the effectiveness of the Early Start Denver Model (ESDM) delivered in a community childcare group setting | 57 children (aged 0–6 years) with a previous ASD diagnosis | See Vivanti et al. (2014) | MSEL, ADOS-Generic, Vineland-II |
| **Zhou et al. (2018)** | Non-randomised controlled trial | To evaluate the effects of a 26-week, high-intensity, parent-implemented Early Start Denver Model (P-ESDM) intervention on developmental outcomes, severity of ASD and parental stress of ASD toddlers | 43 children, (aged 1.5–2.5 years) with a diagnosis of ASD and their parents | See Zhou et al. (2018) | ADOS-2, CSBS |

*Considering the aim of this review, only standardised outcome measures used will be listed. Legend: ASD - autism spectrum disorder; DLD - developmental language disorder; M - mean; SD - standard deviation; SSIS - Social Skills Improvement System; SRS - Social Responsiveness Scale; SSRS - Social Skills Rating System; TASSK - Test of Adolescent Social Skills Knowledge; TL-ALPE - Teste de Linguagem - Avaliação da Linguagem Pré-Escolar; EAC - Escala de Avaliação de Competências Comunicativas; MSEL - Mullen Scales of Early Learning; ADOS - Autism Diagnostic Observation Scale; Vineland - Vineland Adaptative Behavior Scale; CSBS - Communication and Symbolic Behaviour Scale.
